# Gab2 and Gab3 Redundantly Suppress Colitis by Modulating Macrophage and CD8^+^ T-Cell Activation

**DOI:** 10.3389/fimmu.2019.00486

**Published:** 2019-03-18

**Authors:** Zhengqi Wang, Tamisha Y. Vaughan, Wandi Zhu, Yuhong Chen, Guoping Fu, Magdalena Medrzycki, Hikaru Nishio, Silvia T. Bunting, Pamela A. Hankey-Giblin, Asma Nusrat, Charles A. Parkos, Demin Wang, Renren Wen, Kevin D. Bunting

**Affiliations:** ^1^Division of Hem/Onc/BMT, Department of Pediatrics, Aflac Cancer and Blood Disorders Center, Emory University, Atlanta, GA, United States; ^2^BloodCenter of Wisconsin, Milwaukee, WI, United States; ^3^Department of Pathology, Emory University, Atlanta, GA, United States; ^4^Department of Pathology, Children's Healthcare of Atlanta, Atlanta, GA, United States; ^5^Department of Veterinary Science, Pennsylvania State University, University Park, PA, United States; ^6^Department of Pathology, University of Michigan, Ann Arbor, MI, United States

**Keywords:** inflammatory bowel disease, colitis, Grb2-associated binding protein, effector CD8^+^ T-cell, CD4^+^ T-cell, macrophage, intraepithelial lymphocyte

## Abstract

Inflammatory Bowel Disease (IBD) is a multi-factorial chronic inflammation of the gastrointestinal tract prognostically linked to CD8^+^ T-cells, but little is known about their mechanism of activation during initiation of colitis. Here, Grb2-associated binding 2/3 adaptor protein double knockout mice (Gab2/3^−/−^) were generated. Gab2/3^−/−^ mice, but not single knockout mice, developed spontaneous colitis. To analyze the cellular mechanism, reciprocal bone marrow (BM) transplantation demonstrated a Gab2/3^−/−^ hematopoietic disease-initiating process. Adoptive transfer showed individual roles for macrophages and T-cells in promoting colitis development *in vivo*. In spontaneous disease, intestinal intraepithelial CD8^+^ but much fewer CD4^+^, T-cells from Gab2/3^−/−^ mice with rectal prolapse were more proliferative. To analyze the molecular mechanism, reduced PI3-kinase/Akt/mTORC1 was observed in macrophages and T-cells, with interleukin (IL)-2 stimulated T-cells showing increased pSTAT5. These results illustrate the importance of Gab2/3 collectively in signaling responses required to control macrophage and CD8^+^ T-cell activation and suppress chronic colitis.

## Introduction

The healthy gut has strong mechanisms in place to promote tolerance and control inflammation in response to environmental triggers such as infection and bacterial products. When this inflammation is not resolved, chronic uncontrolled inflammation of the intestine results in inflammatory bowel disease (IBD) (1). There are two major types of IBD in humans. Ulcerative colitis is primarily a disease of the colon and rectum, with inflammation and ulceration of mucosa and submucosa. In contrast, Crohn's disease affects the small intestine and colon, typically involving transmural inflammation, ulceration, and fistulae. Human IBD is a complex disorder resulting from the combination of genetic predisposition, environmental triggers, and the loss of integrity of the mucosal immune system. The genetic susceptibility is higher in Crohn's disease than in ulcerative colitis in humans, although these diseases have indistinguishable phenotypes in mouse models of colitis. Intestinal bacteria also play a major role in the pathogenesis of chronic immune mediated intestinal inflammation and certain knockout mice such as IL-10^−/−^ strains require the presence of enteric bacteria in order to initiate colitis (2).

In humans, genome-wide association studies have provided important evidence for genes involved in IBD pathology. Clusters of variants associated with human IBD include loss of epithelial barrier function, increased effector cell responses, changes in translation and metabolism, and immune regulatory cell defects. Numerous animal models of mucosal inflammation have validated several major categories of variants capable of dysregulating immune responses in mice (3, 4). While many of these involve the epithelium and loss of barrier function, less is known about cell intrinsic changes in hematopoietic cells, especially CD8^+^ T-cells which are emerging as important regulators of human IBD (5) but are not well-characterized in this regard. Animal models on the role of T-cells in colitis development have also largely focused on CD4^+^ T-cells. Interestingly, a “guilt-by-association” study by Lee et al. (6) reported on a major connecting node of signaling involving the Grb2 adaptor protein in Crohn's disease. Therefore, in this study we set out to analyze a potential novel linkage between Grb2 and CD8^+^ T-cells.

Although Grb2 has been well-characterized in immune signaling, Grb2-associated binding proteins have not. Grb2-associated binding protein 2 (Gab2) was first cloned in 1998 (7) as the second of 3 members of the family that share a Pleckstrin homology domain and regulate the PI3K-mTOR pathway through interactions with the p85 subunit of PI3K. This pathway was important to study because clinically effective drugs have been generated to members of the pathway and their roles in modulating macrophage and T-cell activation have been described (8–12). It is known that mTOR is a central sensor of nutrient availability in T-cells and that activation of mTOR is a sensitive regulator of metabolism, promoting CD8 maturation (13), T-bet expression, and the CD122 target gene in an IL-15 dependent manner (14). mTORC1 inhibition inhibits T-bet and CD122 expression but preserves memory development by promoting Eomes expression in a dosage-dependent manner (14–17). Since mTORC1 inhibits mTORC2 through negative feedback, selective regulation CD8 T-cell differentiation is achieved (18). Rictor (mTORC2) activity drives glycolytic effector T-cells (19) and Rictor deficiency promotes a memory phenotype (20).

Gabs form signalosomes and interact with additional partners including Grb2 and SHP-2 to bring together positive and negative regulators of immune cell activation, although the physiological significance is not defined. Gab2^−/−^ mice have some impaired cellular functions (21–25) but Gab3^−/−^ mice have no defined phenotypes alone (26) despite high Gab3 expression in several immune cell types (27–29). Most studies of Gab2 function in T-cells have focused on T-cell receptor (TCR) activation in cell lines (30–34). IL-2 and IL-15 signaling through IL-2Rβ results in Gab2 phosphorylation (35). However, it is not known whether Gab2 has any functional role in IL-2/IL-15 signaling.

Interestingly, Gab-like molecular mimicry by Helicobacter pylori CagA virulence factor (36–38) has been consistently inversely related to human IBD (39–41). Gain-of-function of CagA driven gastritis in mice and humans seems to have a protective effect against colitis perhaps through immune evasion (42) raising the possibility that Gab adaptor proteins may have a similar function to maintain normal steady-state immune homeostasis. Therefore, we set out to determine whether Gabs are immune suppressive *in vivo* and to identify the underlying cellular and molecular mechanism. To investigate potential complementary function of these two Gabs, novel Gab2/3^−/−^ mice were generated. A fraction of these double knockout, but not single knockout, mice developed rectal prolapses and suffered from diarrheas driven by macrophages and predominately CD8^+^ T-cells, supporting a novel major redundant role for Gab2/3 in immune cell inactivation required for the suppression of colitis.

## Materials and Methods

### Antibodies and Mice

All antibodies used are listed in Table S1. All animal studies were conducted in compliance with relevant local guidelines, such as the US Department of Health and Human Services Guide for the Care and Use of Laboratory Animals and were approved by the Institutional Animal Care and Use Committees (IACUCs) at Emory University and the BloodCenter of Wisconsin. Gab2^−/−^ mice were generously provided by Dr. Toshio Hirano (Osaka University) and backcrossed 9 generations to C57BL/6J. Gab3^−/−^ mice were generously provided by Dr. Larry Rohrschneider (Fred Hutchinson Cancer Research Center) and backcrossed 11 generations to C57BL/6. Double knockout Gab2/3^−/−^ mice were initially generated by inter-crossing heterozygote Gab2^+/−^Gab3^+/−^ mice and maintained by both heterozygote and homozygote crosses. Animals were housed under a standard day/night cycle with free access to food and water. Progeny were genotyped for Gab2 and Gab3 deletion by PCR and the expected genotype ratios were obtained. Wild-type (WT) C57BL/6J mice (000664), enhanced green fluorescent protein (GFP) transgenic mice (003291), B6 (Cg)-*Rag2*^*tm1.1Cgn*^/J Rag2^−/−^ mice (008449), and B6.SJL-*Ptprc*^*a*^ *Pepc*^*b*^/BoyJ mice (002014) were all from the Jackson Laboratory (Bar Harbor, ME).

### Peripheral Blood Hematology, Endpoints for Euthanasia, and Macroscopic Colon Inflammation Scoring of Mice With Spontaneous Active Colitis

Following puncture of either the facial vein or the retro-orbital venous sinus, WT, Gab2^−/−^, Gab3^−/−^, and Gab2/3^−/−^ mice peripheral blood was collected in a heparinized capillary tube (Thermo Fisher Scientific, Norcross, GA). Mouse hematology was determined using a HemaTrue Hematology Analyzer (Heska, Loveland, CO). Mice showing loss of 25% body weight, major organ failure, rectal prolapse, hind limb paralysis, or clinical or behavioral signs described above for more than 24 h after appropriate intervention (and no response) were euthanized by the CO_2_ method according to the IACUC protocol. Macroscopic colon inflammation score (0–9): Colon weight was scored as follows: 200–275 mg = 0; 276–350 mg = 1; 351–425 mg = 2; 426–550 mg = 3. Colon thickness was scored as follows: none = 0; mild = 1; moderate = 2; severe = 3. Stool consistency was scored as follows: hard = 0; mostly hard but some soft = 1; mostly soft but some hard = 2; diarrhea = 3.

### Lipocalin-2 Assay on Stool Samples

Quantification of stool lipocalin-2 was performed as previously described (43). Briefly, fresh stool samples were collected, weighed, and reconstituted in PBS containing 0.1% Tween 20 at a concentration of 100 mg/ml. Samples were crushed and vortexed to obtain homogenous suspensions. These suspensions were then centrifuged at 12,000 rpm, 4°C for 10 min. The supernatants were collected and used for further analysis. The level of lipocalin-2 was determined using murine lipocalin-2/NGAL DuoSet ELISA kit (R&D Systems, Minneapolis, MN).

### Spontaneous Colitis Histology and Scoring

Histological examination was performed on whole colons of experimental and control mice. Microscopy was performed at room temperature using an Olympus BX41 microscope with an OlymDP21 camera (Olympus America Inc., Center Valley, PA). CellSens imaging software (downloaded from https://www.olympus-lifescience.com) was used for image acquisition. Histological parameters were quantified in a blinded fashion using parameters as previously described (44, 45) with some modification for spontaneous development (developed by TYV and CAP and all 3 pathologists on this study (STB, AN, CAP) trained TYV on morphologic evaluation). Hematoxylin and eosin stained sections (Emory Winship Pathology Core) of Swiss roll mounts of entire mouse colons were assessed for the percentage of the mucosa containing ulceration (0–4), crypt epithelial injury/damage (0–4), and infiltrate leukocytes masses (0–4). The spontaneous disease activity index included the average percentage of these three scoring parameters.

### Dextran Sodium Sulfate (DSS)-Induced Colitis

DSS (molecular mass, 36–50 kDa; MP Biomedicals, Solon, OH) was dissolved in purified water and administered to mice orally at 2.5% weight/volume. Mice were allowed free access to food and drinking water containing DSS from day 0 until day 7, and DSS was withdrawn to allow recovery from colitis for an additional 7 days. Daily clinical assessment of DSS-treated animals included evaluation of stool consistency (hard = 0; soft = 2; diarrhea = 4), detection of blood in stool (negative = 0; positive = 2; macroscopic = 4), and body weight loss measurements (no loss = 0; 1–5% loss = 1; 5–10% loss = 2; 10–20% loss = 3; >20% loss = 4). DSS-treated recipient mice individual scores were attributed for each one of these parameters and a disease activity index ranging from 0 to 4 was calculated by averaging all 3 scores. Remaining mice were euthanized on day 14 and colons were collected for further analysis.

### BM Transplantation to Generate Hematopoietic Chimeras

BM cells were isolated from 8 to 12 week old WT and Gab2/3^−/−^ mice (CD45.2^+^) and were then transplanted by intravenous injection of about 1 × 10^7^ cells into lethally-irradiated (1,100 rads; Cs^137^ source) BoyJ (CD45.1) recipient mice at a ratio of 1–2 donor equivalents (both hind limbs) per five recipients. Body weights were documented before and after transplantation and monitored bi-weekly for weight loss. Mice were monitored for 11 weeks and colons were collected for histological analysis. In some experiments, BM isolated from 8 to 12 week old BoyJ mice were transplanted into lethally irradiated either WT or Gab2/3^−/−^ recipient mice. Three months after transplantation, the percentage of donor engraftment was analyzed by flow cytometry using mouse peripheral blood following staining with antibodies to multiple hematopoietic lineage markers (CD8, CD4, B220, Gr-1, Ter119). Colons were also collected for histological analysis.

### Newborn Injections

Pups generated from Gab2^+/−^Gab3^−^ × Gab2^−/−^Gab3^−/−^ mice were injected intraperitoneally with 1 × 10^7^ CD45.1 congenic positive or GFP transgenic mouse BM cells 1–2 days after birth. At 4 weeks of age, the newly weaned mice were genotyped for Gab2 and Gab3. At 16 weeks following injection, the mice were analyzed by flow cytometry of the peripheral blood leukocytes for engraftment with CD45.1 congenic or GFP positive donor cells. The colon was also collected for histology analysis.

### BM-Derived Macrophage Culture

BM-derived macrophages (BMDM) were prepared using 8–12 week old WT, Gab2^−/−^, Gab3^−/−^, and Gab2/3^−/−^ mice. Briefly, mice were sacrificed and tibia and femurs were collected. Using a 22-gauge needle marrow was flushed and resuspended in PBS containing 2% FBS. Samples were centrifuged at 1,200 rpm for 8 min, 4°C, and the supernatant was removed. Red blood cells were lysed using a sterile-filtered (0.22 μm) hypotonic solution (155 mM NH_4_Cl, 12 mM NaHCO_3_, 0.1 mM EDTA) and cell mixtures were filtered using a 40 μm cell strainer followed by centrifugation as described above. Cells were resuspended in complete Dulbecco's Modified Eagle Medium (DMEM). Cells were supplemented with 30% L929 conditioned media and incubated for 6 days. Additional feedings were performed at day 3 of culture. All experiments in this study were conducted using CD11b^+^/F4/80^+^ mature macrophages as confirmed by flow cytometry.

### T-cell Expansion and IL-2 Stimulation

Twenty-four well plates were coated in PBS with 1 μg/ml anti-CD3 and 0.5 μg/ml anti-CD28 at 4°C overnight, and then the plates were used for cell culture after washing and blocking. The fresh mouse spleen single cell suspension was adjusted to a concentration of 1 × 10^6^ cells/mL in RPMI 1640 with 10 ng/ml of IL-2, and added into the pre-coated plates. After 3 days culture, cells were harvested and washed with RPMI 1640, and re-cultured in IL-2 (10 ng/mL) at 2 × 10^5^ cells/mL in new plates for another 3 days. Cells were counted and stained with different T-cell surface markers (CD4, CD8, CD62L, and CD44) at day 3 and day 6 post culture to check the expansion of T-cells. The IL-2 cultured cells were further collected, washed with PBS, and then starved in complete RPMI 1640 media containing 1% FBS for 5 h. After 5 h starvation, cells were left unstimulated or re-stimulated with 10 ng/mL of IL-2 for 5, 15, or 40 min. Protein samples were collected and Western blot was performed as described in the section Materials and Methods.

### RNA Isolation and Real-Time Quantitative PCR

To examine the effects of Gabs on mRNA expression of inflammatory cytokines in WT, Gab2^−/−^, Gab3^−/−^, and Gab2/3^−/−^ BMDM cells, total cellular RNA was extracted from 3 × 10^6^ cells using Trizol reagent (Invitrogen, Carlsbad, CA, U.S.A) according to the manufacturer's protocol and cDNA was synthesized from 1.0 μg of RNA using the high capacity cDNA reverse transcriptase kit protocol (Applied Biosystems, Foster City, CA). Real-time PCR was conducted using iQ SYBR Green Supermix (Bio-Rad, Hercules, CA) and amplification was performed on a 7500 Real-time PCR instrument (Applied Biosystems, Foster City, CA). Inflammatory target genes were examined and normalized to glyceraldehyde-3-phosphate dehydrogenase (GAPDH).

### Western Blot Analysis

Cells were treated with 100 ng/ml of LPS for the indicated time periods. Whole cell lysates were collected from BMDM and solubilized proteins were separated by 10% SDS-PAGE, transferred to PVDF membrane (Bio-Rad, Hercules, CA), and detected by immunoblotting with specific primary antibodies and IRDye 800CW or IRDye 680RD secondary antibody with the Odyssey Imaging System (LI-COR Biosciences, Lincoln, NE).

### Cytokine Protein Production Measured by Luminex Assay

Briefly, 1 × 10^6^ BMDMs were plated and treated for 12 h with 100 ng/ml LPS (γ-irradiated, BioXtra, from E. coli O111:B4, Sigma). After centrifugation, supernatants were collected, frozen, and used for Luminex assay. To measure TNFα, and IFN-γ protein secretion, Luminex assay was performed using the Invitrogen Luminex Cytokine Mouse 10-plex Panel following the manufacturers' protocol (Invitrogen, Carlsbad, CA, USA) and using the Luminex 100 system (Luminex Corp., Austin, TX).

### Colon Organ Tissue Culture

Freshly isolated mouse colon was washed twice (15 min each) with PBS, 3 mm length colon was cut, then weighed and placed into 48-well plate with 500 μl IMEM with 5% FBS and cultured for 24 h. The supernatant was collected, frozen, and used for Luminex assay using the Invitrogen Luminex Cytokine Mouse 10-plex Panel following the manufacturers' protocol (Invitrogen, Carlsbad, CA).

### Isolation of Macrophages, IELs, and LPLs From Mouse Colon

Mouse colon macrophages were isolated using the protocol (46). Briefly, the colon was cut open longitudinally using scissors and fecal contents were washed off along with mucus from the colon lumen using Ca^2+^/Mag^2+^-free (CMF) PBS at room temperature. Then, the colon was cut into approximately 1.5 cm pieces and placed into 50 mL conical tubes with 30 mL of pre-warmed CMF Hank's balanced salt solution (HBSS) with 5% FBS and 2 mM EDTA, and shaken at 250 rpm for 20 min at 37°C, followed by a repeated wash. Colon pieces (1.5 cm) were recovered and washed, followed by mincing and digestion in 20 mL of collagenase solution (1.5 mg/mL type VIII collagenase dissolved in pre-warmed CMF HBSS/FBS with 40 μg/mL of DNase I) at 200 rpm for 13–15 min at 37°C. It was filtered through a 100 μm cell strainer directly into a 50 mL conical tube and centrifuge at 1,500 rpm for 5 min at 4°C and the cell pellet was resuspended in ice-cold CMF HBSS with 5% FBS. Finally CD45, CD11c, MHC II, CD11b, and F4/80 antibody staining was performed, followed by multi-color flow cytometry. Intraepithelial lymphocytes (IELs) and lamina propria lymphocytes (LPLs) from mouse colon were isolated using a Lamina Propria Dissociation Kit (Miltenyi Biotec Inc., Auburn CA) following the user's manual.

### Flow Cytometry Analyses of Tissue Immune Cell Numbers

To assess *in vivo* macrophage numbers, digested cells and splenocytes were stained with fluorescence conjugated antibodies against MHC II, CD45, F4/80, and CD11b (eBioscience, San Diego, CA). LIVE/DEAD staining (Thermo Fisher Scientific) was used to assay for cell viability determination at 405 nm excitation. For intracellular cytokine staining, freshly isolated IELs and splenocytes were incubated with 50 ng/ml PMA (Sigma), 750 ng/ml ionomycin (Sigma, St. Louis, MO) and 5 μg/ml Brefeldin A (Biolegend, San Diego, CA) at 37°C for 4 h. Cells were first collected for surface staining with anti-CD8-BV650 (Biolegend, San Diego, CA), anti-CD62L-BV605 (Biolegend, San Diego, CA), anti-CD4-PE Cy7, and anti-CD44-APC (Thermo Fisher Scientific, Waltham, MA) antibodies, and then fixation and permeabilization were performed following the instructions from BD Cytofix/Cytoperm Fixation/Permeabilization Solution Kit (BD Biosciences, San Jose, CA). Intracellular staining for cytokines was detected with anti-IFN-γ-PE, anti-TNF-α-FITC, and anti-IL-17-Percp Cy5.5 antibodies (Biolegend, San Diego, CA). Data were collected using a BD LSRII flow cytometer (BD Biosciences, San Jose, CA) and analyzed using FlowJo software (FlowJo, LLC, Ashland, OR).

### Adoptive Transfer of T-Cells and BMDMs

Details of antibodies used for T-cell sorting are listed in Table S1. WT or Gab2/3^−/−^ spleens obtained from 8 to 12 week old mice were used for T-cell isolation. CD4^+^ or CD8^+^ cells were isolated by using either CD4^+^ T-cell isolation kit or CD8^+^ T-cells isolation kit (Miltenyi Biotec, Sunnyvale, CA). Naïve CD4 T-cells (CD4^+^CD45RB^high^) or naïve CD8^+^ T-cells (CD44^−^CD62L^+^CD8^+^) were FACS-sorted. Naïve CD4^+^ (8 × 10^5^) or naïve CD8^+^ (4 × 10^5^) T-cells in 250 μL 2% FBS in PBS were injected into 8–12 week old Rag2^−/−^ mice by intraperitoneal injection. For some experiments, 8–12 week old Rag2^−/−^ mice were injected with 1 × 10^6^ BMDMs in 250 μL 2% FBS in PBS from either WT or Gab2/3^−/−^ by IP injection first. Twenty-four hours later, those mice were transferred with 8 × 10^5^ FACS sorted WT naïve CD4^+^ T-cells, Mice were monitored daily, weighed weekly, and euthanized at the end of 8 weeks after the T-cell transfer or earlier if meeting euthanasia criteria as described in this section. Colon length and weight were measured and colons were prepared for histology analysis.

### Statistical Analyses

Student's two tailed *t*-test was used to calculate *P*-values using the statistical function in the Microsoft excel and *F*-test was used to determine that the two groups had equal or unequal variances. For survival analysis, the log rank test was used to calculate *P*-values (SAS 9.4, SAS Institute, Cary, NC). For all statistical analyses, *P* < 0.05 were considered to be significant.

## Results

### Gab2 and Gab3 Have Redundant Functions in Suppression of Spontaneous Colitis

To investigate whether Gab proteins play a redundant role in regulating the immune system, WT, Gab2^−/−^, Gab3^−/−^, and Gab2/3^−/−^ mice were generated and monitored for at least 8 weeks for physiological changes and/or infection. Gab2/3^−/−^ mice have modest reductions in peripheral blood hematology relative to WT and single knockout mice (Table 1).

**Table 1:** Peripheral blood hematology of Gab2^−/−^, Gab3^−/−^, and Gab2/3^−/−^ mice.

	**WBC (10^**3**^/μL)**	**LYM (10^**3**^/μL)**		**MONO (10^**3**^/μL)**	**GRAN (10^**3**^/μL)**	**% LYM**	**% MONO**	**% GRAN**
WT (*N* = 37)	8.78 ± 2.28	7.14 ± 1.87		0.51 ± 0.19	1.13 ± 0.66	81.6 ± 6.4	5.2 ± 1.3	13.3 ± 5.3
Gab2^−/−^ (*N* = 8)	8.26 ± 2.16	7.13 ± 2.10		0.36 ± 0.09^**^	0.78 ± 0.07^**^	85.6 ± 3.8^*^	4.0 ± 1.0^*^	10.4 ± 3.0^*^
Gab3^−/−^ (*N* = 8)	8.84 ± 1.79	7.55 ± 1.64		0.46 ± 0.11	0.83 ± 0.17^*^	85.4 ± 2.3^**^	4.5 ± 0.9	10.1 ± 1.5^**^
Gab2/3^−/−^ (*N* = 16)	6.88 ± 2.65^*^	5.89 ± 2.59		0.35 ± 0.08^***^	0.63 ± 0.20^***^	82.6 ± 11.0	5.0 ± 1.8	12.4 ± 9.3
	**HCT (%)**	**MCV (fl)**	**RDWa (fl)**	**RDW %**	**HGB (g/dL)**	**MCHC (g/dL)**	**MCH (pg)**	**RBC (10**^**6**^**/μL)**
WT (*N* = 37)	47.2 ± 3.1	46.1 ± 1.4	31.3 ± 4.3	20.8 ± 2.9	16.5 ± 1.2	34.9 ± 1.2	16.1 ± 0.6	10.2 ± 0.7
Gab2^−/−^ (*N* = 8)	48.6 ± 1.8	44.2 ± 0.6^***^	24.0 ± 0.4^***^	17.2 ± 0.4^***^	16.5 ± 0.5	34.0 ± 0.4^***^	15.0 ± 0.2^***^	11.0 ± 0.4^***^
Gab3^−/−^ (*N* = 8)	44.4 ± 1.9^**^	45.2 ± 1.1	24.7 ± 0.8^***^	17.1 ± 0.4^***^	15.0 ± 0.5^***^	33.8 ± 0.4^***^	15.2 ± 0.4^***^	9.8 ± 0.5
Gab2/3^−/−^ (*N* = 16)	45.9 ± 3.2	45.6 ± 2.5	27.0 ± 3.1^***^	18.5 ± 1.0^***^	15.7 ± 1.1^*^	34.2 ± 0.8^*^	15.6 ± 0.7^*^	10.1 ± 0.9

* P < 0.05;

** P < 0.01;

*** P < 0.001;

*unpaired t-test relative to WT; numbers of mice are indicated in the table. WBC, white blood cells; LYM, lymphocytes; MONO, monocytes; GRAN, granulocytes; HCT, hematocrit; MCV, mean corpuscular volume; RDW, Red cell distribution width; HGB, hemoglobin; MCHC, mean corpuscular hemoglobin concentration; MCH, mean corpuscular hemoglobin; RBC, red blood cells*.

**Figure 1:**
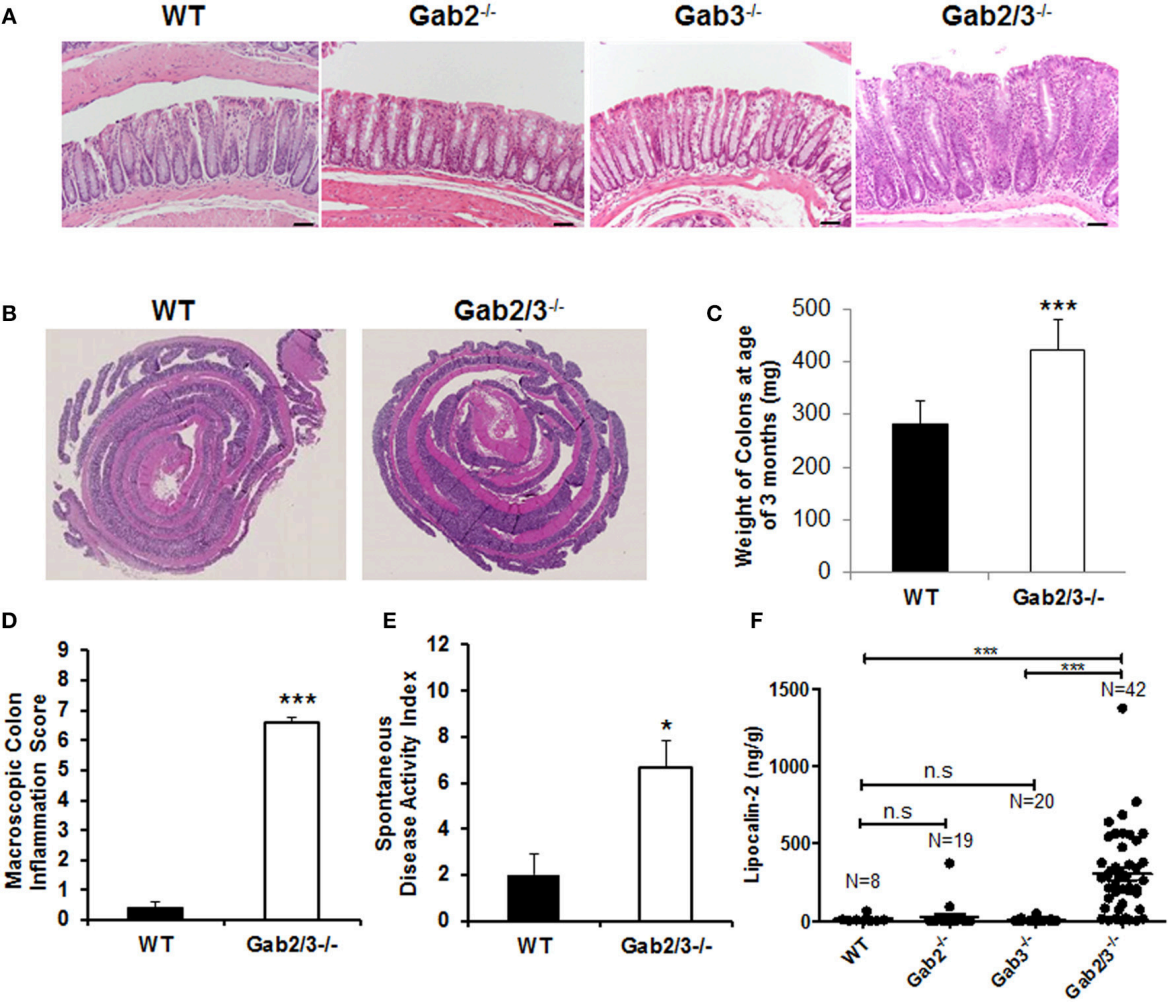
Development of spontaneous colitis in Gab2/3^−/−^ but not Gab2^−/−^ or Gab3^−/−^ mice. **(A)** Representative colon histology from a total of WT, *N* = 3; Gab2^−/−^, *N* = 3; Gab3^−/−^, *N* = 3, Gab2/3^−/−^, *N* = 7 was assessed in WT, Gab2^−/−^, Gab3^−/−^, and Gab2/3^−/−^ mice at 8–12 weeks of age. Bar = 50 μm. **(B)** Representative Swiss roll mounts displayed severe hyperplasia and increased thickness in Gab2/3^−/−^ colons. **(C)** Colon weights at age 3 months were measured for both WT and Gab2/3^−/−^. Data are shown as mean ± standard deviation (SD) (unpaired *t*-test, *N* = 10 for WT, *N* = 11 for Gab2/3^−/−^, ^***^P < 0.001). **(D)** Macroscopic colon inflammatory scores for WT and Gab2/3^−/−^ mice. Scoring colons was assessed by the average of colon weights, thickness, and stool consistency. Data are shown as the mean ± SD (unpaired *t*-test, *N* = 10 for WT, *N* = 11 for Gab2/3^−/−^, ^***^P < 0.001). **(E)** A composite spontaneous disease activity index of WT and Gab2/3^−/−^ mice. Swiss roll mounts of WT and Gab2/3^−/−^ colons were scored for the presence of crypt abscesses, crypt architectural distortion, and hyperplasia. Data are shown as mean ± SD (unpaired *t*-test, *N* = 3 for WT, *N* = 6 for Gab2/3^−/−^, ^*^P < 0.05). **(F)** Stool samples were collected from mice and Lcn-2 enzyme was analyzed by ELISA based assay with the number of mice in each group indicated on the figure. (^***^P < 0.001; unpaired *t*-test).

As early as 8 weeks of age, rectal prolapse was observed in Gab2/3^−/−^ mice and approximately 1/3 of the mice developed rectal prolapse (50/136) compared to none detected in control mice as determined during a representative 15-month observation period. Treatment with antibiotic at breeding or weaning stages ameliorated rectal prolapses indicating that resident microbiota are essential for the etiology of this experimental colitis (data not shown). Since diarrhea, intestinal mass/tumor, or colitis could increase the incidence of rectal prolapse in mice, colonic tissue was analyzed. Inflammation (Figure 1A) and muscular hypertrophy (Figure 1B) was observed on histology analysis in Gab2/3^−/−^ mouse colon tissue compared to WT, Gab2^−/−^, and Gab3^−/−^ control mice. The weights of the Gab2/3^−/−^ colons were increased as well as the inflammation score and the spontaneous disease activity index (0–12 scale) (Figures 1C–E) compared with WT mice.

Interestingly, in addition to colon pathology, serum levels of amylase and globulin enzymes were elevated and albumin levels were decreased relative to WT resulting in a reduced albumin/globulin (A/G) ratio known to be associated with chronic inflammatory disease, including pancreatitis (Table S2). The pathology was tissue-specific since no gross abnormalities were observed outside of the gastrointestinal tract. Lipocalin-2 (Lcn-2) protein is a component of the innate immune response whose expression has been shown to be upregulated during infection. Fecal Lcn-2 has been demonstrated by others to be significantly increased in DSS colitis models potentially serving as a sensitive and non-invasive approach to detect intestinal inflammation (43). To determine whether Lcn-2 would help to better predict colitis development, stool samples were collected and extremely elevated levels of Lcn-2 were observed in about 1/3 of the Gab2/3^−/−^ mice compared to the very low expression in WT mice (Figure 1F). A comparison of WT, Gab2^−/−^, Gab3^−/−^, and Gab2/3^−/−^ mice showed that maximal Lcn-2 increase was observed in double knockout mice (average of 26-fold), whereas mice with rectal prolapse had high Lcn-2 levels, but high Lcn-2 levels did not predict rectal prolapse.

**Figure 2:**
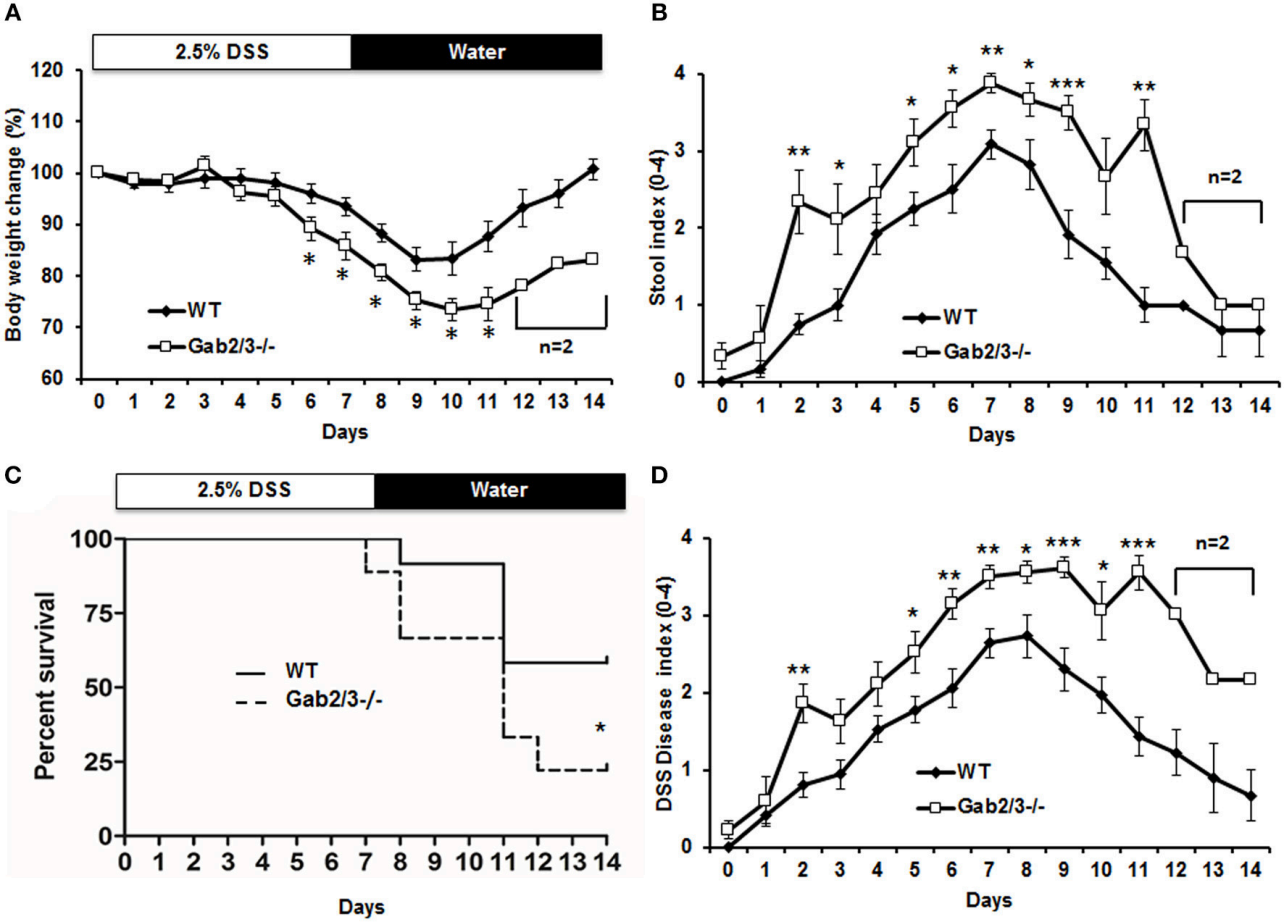
DSS-induced colitis is more severe in Gab2/3^−/−^ mice. WT (*N* = 12) and Gab2/3^−/−^ (*N* = 9) mice were assessed following 2.5% DSS treatment for 7 days and then recovery on water for 7 days. **(A)** Body weight changes were monitored daily and were calculated as percent of the initial body weight at day 0. **(B)** Stool was assessed daily for consistency characterized as hard, soft, or diarrhea and for the presence of blood. **(C)** The percent survival of DSS-treated WT and Gab2/3^−/−^ mice. Log rank test was used to calculate *P*-values (^*^P < 0.05). **(D)** Disease activity index [DAI (47)] scores measured by average of body weight loss, stool consistency, and bloody feces. For a, b, and d, data are shown as the mean ± standard error (SE). Unpaired *t*-test, WT from Day 0–11 (*N* = 7–12), Day 12–14 (*N* = 3); Gab2/3^−/−^ from Day 0–10 (*N* = 6–9), Day 11 (*N* = 3), then Day 12–14 (*N* = 2). ^*^P < 0.05, ^**^P < 0.01, ^***^P < 0.001.

Although Gab2/3^−/−^ mice developed colitis-like phenotypes spontaneously, there was variation in controlling the rate of disease development as well as the severity of inflammation with age. To control for such variation in the spontaneous mouse model, age-matched young asymptomatic mice were orally administered DSS in the drinking water. DSS at 2.5% was given for 7 days followed by a 7 day recovery period. Clinical features of colitis, such as alterations in the intestines were assessed by endoscopy (Figure S1). Body weights and stool consistencies were monitored daily and scoring criteria were calculated in Gab2/3^−/−^ mice (Figures 2A,B) compared to WT mice. Gab2/3^−/−^ mice were extremely sensitive to DSS, resulting in death of 80% of Gab2/3^−/−^ mice by day 14 compared to only 40% of WT mice (Figure 2C). Stool samples were collected during DSS treatment and increases in Lcn-2 were observed on day 3 suggesting increased sensitivity to treatments compared to WT mice (Figure S2). In addition, the DSS disease activity index (0–4 scale; averaged) showed faster onset of disease (Figure 2D) in Gab2/3^−/−^ mice compared to WT mice. Histology measures showed increased inflammation in Gab2/3^−/−^ mice on day 7 (Figure S3A) and day 14 (Figure S3B) relative to WT mice. Disease activity was assessed by scoring as described in the Methods. Greater ulceration, proliferative atypia, and goblet cell loss was observed in Gab2/3^−/−^ mice (Figure S4) compared to WT mice. Altogether, these results suggest that Gab2/3^−/−^ mice are more susceptible to colitis development than WT mice.

### Colitis Development in Gab2/3^−/−^ Mice Is Hematopoietic Cell-Initiated and Associated With Increased Gut Cytokines and T-Cell Invasion

**Figure 3:**
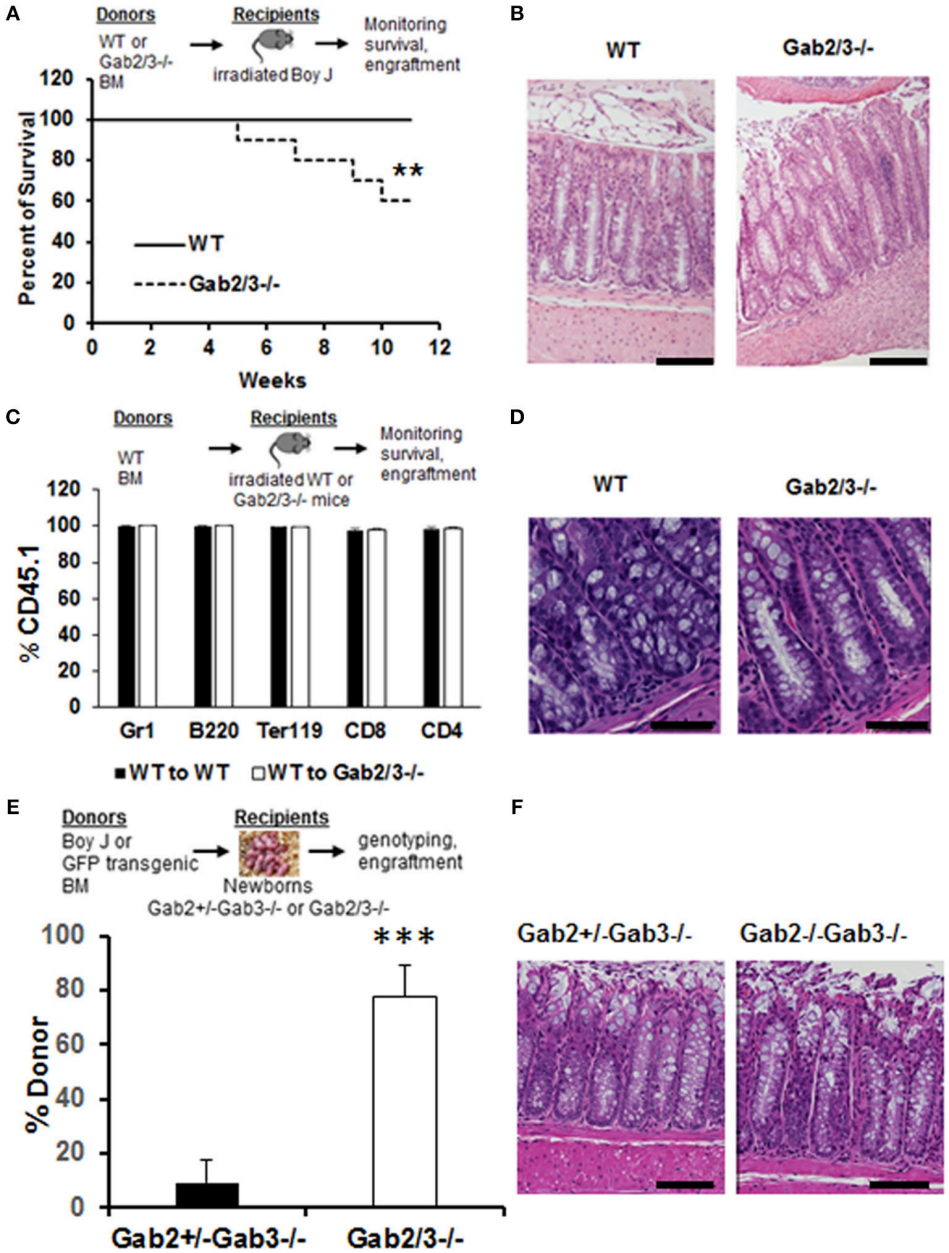
BM from Gab2/3^−/−^ mice induces colitis in WT transplant recipients while reciprocal WT BM transplanted into Gab2/3^−/−^ mice protects from colitis. **(A)** Diagram of WT or Gab2/3^−/−^ BM transplantation into lethally-irradiated BoyJ recipients. Percentage of survival is shown as a Kaplan-Meier survival curve. Log rank test was used to calculate *P*-values (^**^P < 0.01). BM cells isolated from WT or Gab2/3^−/−^ mice were injected into lethally-irradiated BoyJ mice (approximately 1 × 10^7^ cells/recipient). The results shown are the combination of 3 independent experiments with *N* = 15 for each group of mice. **(B)** Representative histology of WT and Gab2/3^−/−^ BM transplanted mouse colon. Bar = 50 μm. **(C)** Diagram of WT BM (CD45.1) transplantation into lethally-irradiated WT or Gab2/3^−/−^ recipients (CD45.2). The percentage of donor CD45.1 engraftment in the peripheral blood of WT BM transplanted WT or Gab2/3^−/−^ mice. Experiments were repeated twice (*N* = 10 mice total for each group). **(D)** Representative normal colon histology in WT or Gab2/3^−/−^ mice engrafted with WT BM donor cells. Bar = 100 μm**. (E)** Diagram of WT (CD45.1) or GFP transgenic BM transplantation into newborn mice on day 1 or 2 and mice were genotyped after weaning and donor engraftment was checked 16 weeks following transplantation. The percentage of donor engraftment in the peripheral blood of newborn recipients. *One* litter was injected with 1 × 10^7^ BoyJ BM cells and the other litter was injected with 1 × 10^7^ GFP transgenic BM cells. Totals of Gab2^+/−^Gab3^−/−^ *N* = 9; Gab2^−/−^Gab3^−/−^, *N* = 4 mice (C57BL/6 background) were obtained and analyzed for evidence of long-term donor engraftment with CD45.1 congenic or GFP positive donor cells 16 weeks following injection. Data shown are the mean ± SD, ^***^P < 0.001; unpaired *t*-test. **(F)** Representative colon histology of Gab2^+/−^Gab3^−/−^ and Gab2^−/−^Gab3^−/−^ mice. Mice were euthanized at the age of 5 months and colon histology was examined. Bar = 50 μm.

To understand the cellular mechanisms of colitis development in Gab2/3^−/−^ mice, BM chimeras were generated using WT or Gab2/3^−/−^ donors on the C57BL/6J background into lethally-irradiated WT BoyJ (CD45.1 congenic) recipient mice. After immune reconstitution by donor BM cells, chimeric mice were monitored for 11 weeks. High levels of donor CD45.2 multilineage engraftment were observed in both WT and Gab2/3^−/−^ transplanted recipients 8 weeks after transplantation (Figure S5). Of the Gab2/3^−/−^ BM transplant recipients, 6/15 died whereas none of the WT BM recipients died (Figure 3A). Rag2^−/−^ recipients were also tested and all mice receiving Gab2/3^−/−^ BM transplant died within 11 weeks after transplantation, whereas recipients of WT BM were all healthy with normal colon histology (data not shown). Susceptible recipients of Gab2/3^−/−^ BM showed a colitis phenotype (Figure 3B) resulting in forced euthanasia due to body weight decreases ≥25%. Interestingly, multiple ulcerations were present in most of the colon proximal region, with extensive epithelial damage, transmural inflammation, and adenocarcinoma in some recipients (Figure S6). Inversely, when WT donor BM cells were transplanted into lethally-irradiated Gab2/3^−/−^ mice, engraftment with donor-derived WT cells (CD45.1^+^) was observed at a very high level (Figure 3C). Gab2/3^−/−^ recipients did not develop disease and maintained normal colon epithelial crypt architecture (Figure 3D). Furthermore, in order to rule out potential radiation-induced damage to colon tissue, unconditioned Gab2/3^−/−^ newborn recipients were transplanted and also showed a high percentage of donor engraftment after weaning (Figure 3E) and upon euthanasia at 5 months, 4/4 of the injected Gab2/3^−/−^ mice showed normal colon histology (Figure 3F). With either lethal irradiation or no irradiation, both showed that engraftment of WT BM prevents disease. Therefore, colitis resulting from Gab2/3 deficiency is hematopoietic cell-derived.

**Figure 4:**
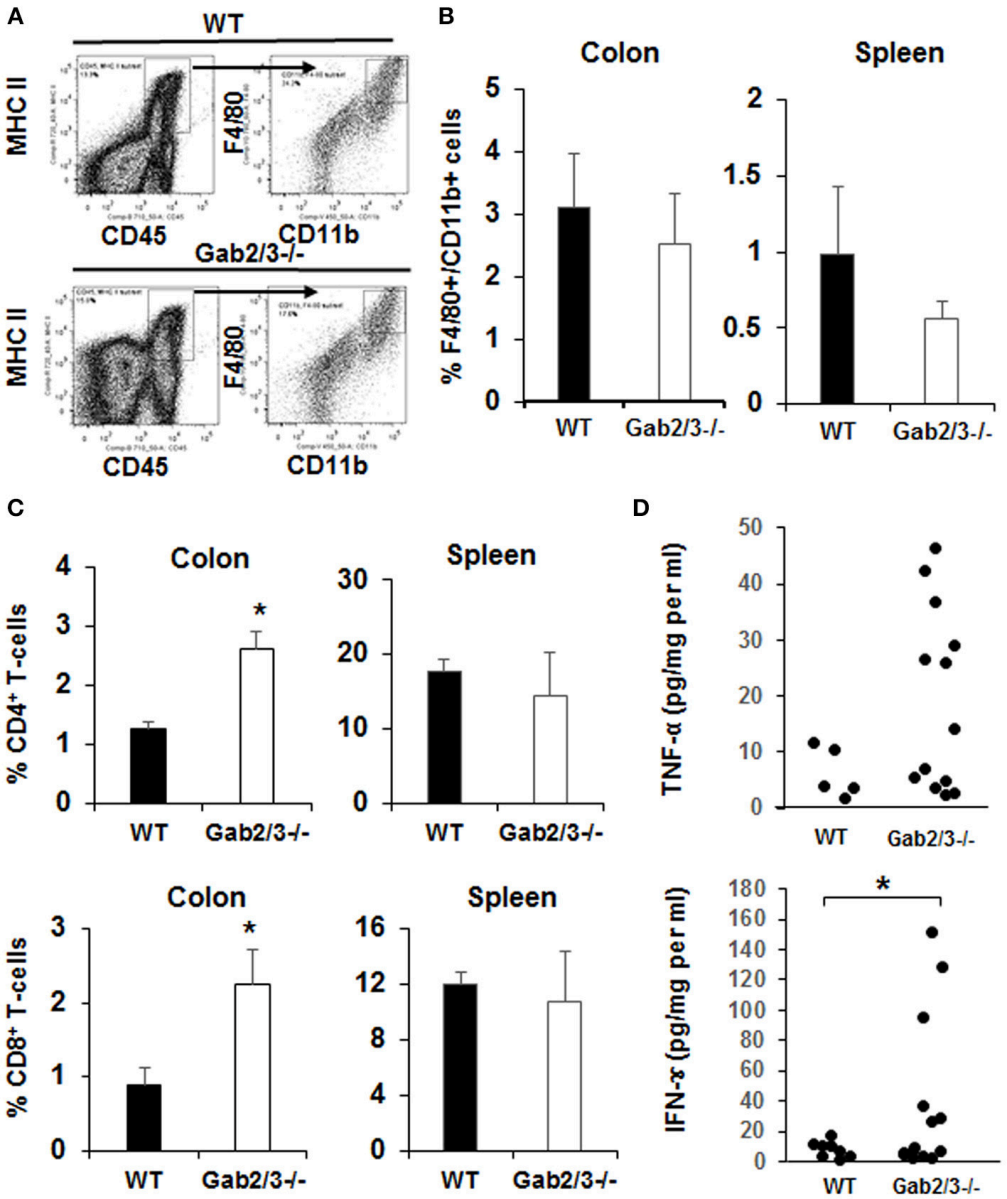
Gab2/3^−/−^ mice have increased macrophage and T-cell invasion of the colon and production of pro-inflammatory cytokines from colon extracts. **(A)** The flow cytometry gating strategy for F4/80 and CD11b^+^ cells is shown. Dead cells were excluded using the LIVE/DEAD fixable aqua dead cells stain kit (Invitrogen, Carlsbad, CA) and subsequent analyses were gated on live cells. Antigen presenting cells (APCs) were selected by MHC II and CD45 double staining, and F4/80 and CD11b cells were gated on APCs. Data were acquired with a LSRII flow cytometer (BD Biosciences, USA) and analyzed with FlowJo Software. **(B)** The percentages of F4/80 and CD11b double positive cells in the colon and spleen are shown as the mean ± SD (*N* = 4). **(C)** The percentage of total CD4^+^ and CD8^+^ cells in lamina propria lymphocytes (LPL) after colon digestion with collagenase or in mouse splenocytes. Data are shown as the mean ± SD (*N* = 4, ^*^P < 0.05; unpaired *t*-test). **(D)** Freshly isolated mouse colon was used for colon organ culture with a 24-h collection time in IMEM with 5% FBS. Cytokines in cultured medium were measured using the mouse multiplex cytokine assay kit on a Luminex 100 instrument (*N* = 7, duplicated measurement). For comparison of WT vs. Gab2/3^−/−^ groups; TNF-α *P* = 0.06 and for IFNγ ^*^P = 0.02; unpaired *t*-test.

To identify specific immune cells which may have contributed to colitis in Gab2/3^−/−^ mice, flow cytometry analyses were performed on colon, and spleen. Colon digestion was performed as described (46) and the antigen-presenting cell was identified by CD45 and MHC II co-expression. These cells were then analyzed for CD11b and F4/80 expression (Figure 4A). There was no change in the percentage of F4/80^+^CD11b^+^ macrophages observed in the spleen or colon (Figure 4B) between Gab2/3^−/−^ and WT groups. However, significantly increased percentages of CD4^+^ and CD8^+^ T-cells were observed in the colon but no differences in overall splenic T-cells was observed (Figure 4C) between Gab2/3^−/−^ and WT. Comparable changes were observed in absolute numbers of cells (Figure S7). This result suggested that T-cells play a role in colitis development. Colon organ tissue from WT, or Gab2/3^−/−^ mice with active disease, was cultured for 24 h and cytokines in the cultured medium were analyzed by Luminex assay. Inflammatory cytokines including IFNγ and TNFα were observed in Gab2/3^−/−^ tissue cultures (Figure 4D). Notably, increases relative to WT mice were only observed in active disease (rectal prolapse and/or high Lcn-2). All mice utilized for these experiments had active disease but some of them developed rectal prolapse and others did not.

### Gab2/3^−/−^ Macrophages Are Pro-Inflammatory and Promote Colitis Following Adoptive Transfer into Immune-Deficient Hosts

**Figure 5:**
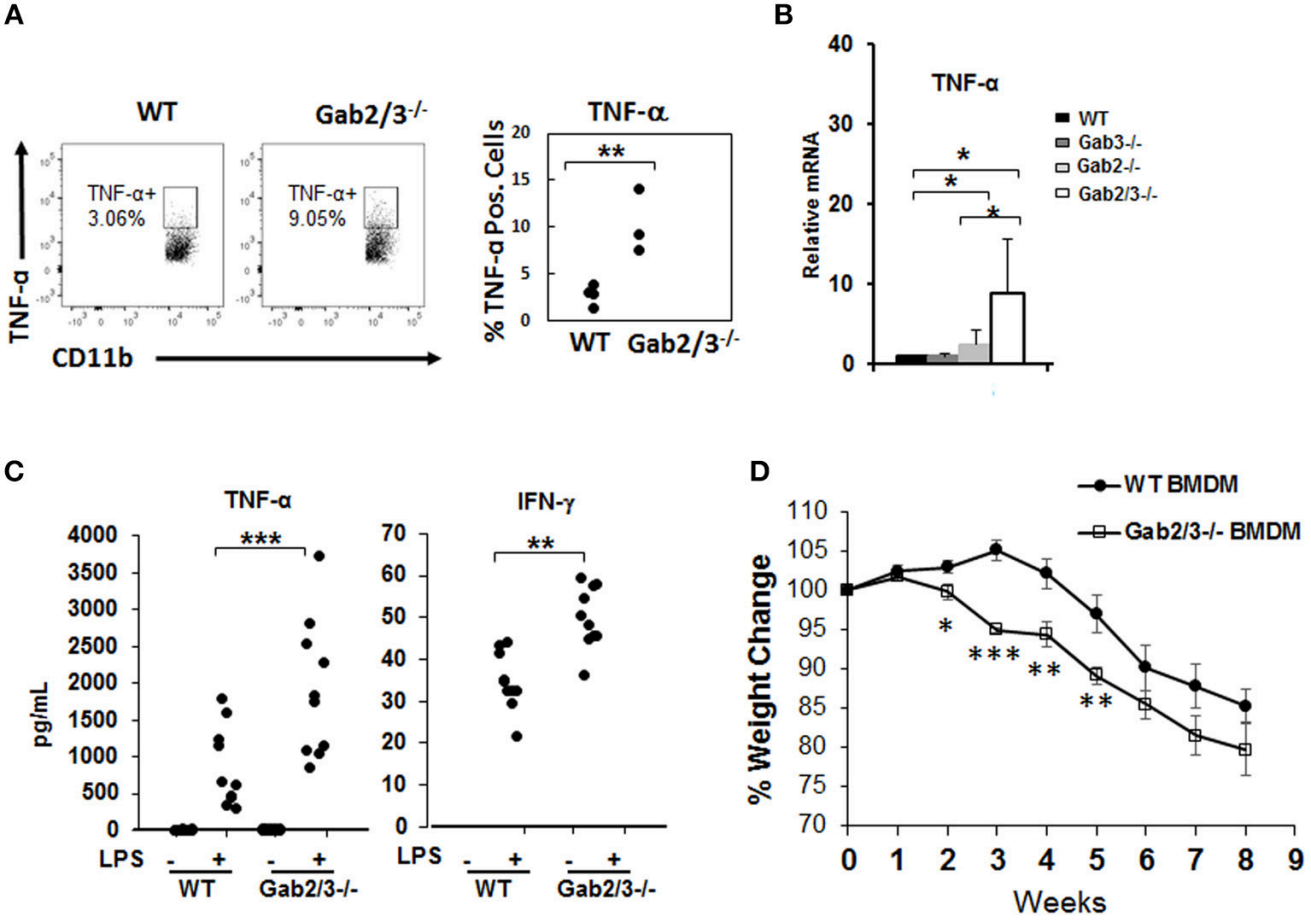
Increased induction of pro-inflammatory cytokines from unstimulated Gab2/3^−/−^ BMDMs. **(A)** Freshly isolated BM from WT or Gab2/3^−/−^ mice (high Lcn-2 level) were analyzed by intracellular flow cytometry using CD11b and F4/80 markers to identify macrophages and TNF-α antibody was used to detect cytokine-expressing macrophages (unpaired *t*-test, ^**^P < 0.01). **(B)** BMDMs were analyzed for TNF-α mRNA levels by qRT-PCR. Both WT and Gab3^−/−^ BMDM, *N* = 7; Gab2^−/−^ BMDM, *N* = 5 and Gab2/3^−/−^ BMDM, *N* = 3; ^*^P < 0.05, unpaired *t*-test. **(C)** BMDMs were treated with or without LPS for 12 h and supernatant was collected to detect protein levels of TNF-α and IFN-γ by Luminex assay. The results shown represent 3 or more independent experiments. (^**^P < 0.01; ^***^P < 0.001; unpaired *t*-test). IFNγ levels were undetectable in unstimulated BMDMs. **(D)** WT or Gab2/3^−/−^ LPS-activated BMDM cells (1 × 10^6^ each) were transferred into each Rag2^−/−^ mouse by IP injection and 24 h later each mouse also received 8 × 10^5^ sorted WT naïve CD4^+^ T-cells. The weight change was monitored weekly and is shown as the mean ± SE for *N* = 11 for WT, *N* = 8 for Gab2/3^−/−^ groups. Relative to WT, the time points for Gab2/3^−/−^ with a significant change are indicated on the figure (^*^P < 0.05; ^**^P < 0.01; ^***^P < 0.001, unpaired *t*-test). The results are representative from two independent experiments.

Increased inflammatory cytokines can be derived from macrophages which are key cells of the innate immune system with TLR4 receptors highly expressed on the cell surface. To investigate the role of macrophages in colitis, freshly isolated macrophages from WT and Gab2/3^−/−^ BM cells and BMDMs from WT, Gab2^−/−^, Gab3^−/−^, and Gab2/3^−/−^ mice were analyzed. The role of Gab2/3 was first examined on cytokine production by macrophages. Macrophages were identified by CD11b and F4/80 co-staining and were analyzed for TNFα and IFNγ expression as measured by intracellular flow cytometry. Primary Gab2/3^−/−^ macrophages had elevated percentages (but not absolute numbers) of TNFα positive cells when compared to WT cells (Figure 5A). Second, BMDMs were cultured *in vitro* from Gab2/3^−/−^ mice expanded and co-expressed CD11b and F4/80 (Figure S8). Analysis of culture-expanded Gab2/3^−/−^ BMDMs screened for expression of pro-inflammatory cytokines by qRT-PCR showed elevated TNFα when compared to control or Gab2^−/−^ or Gab3^−/−^ cells (Figure 5B). No change was observed for IFNγ expression (data not shown). After LPS treatment, secreted protein levels of both TNFα and IFN-γ (Figure 5C) were significantly higher in BMDM conditioned media obtained from cells derived from Gab2/3^−/−^ mice with rectal prolapse compared with WT.

The role of Gab2/3 in macrophages during colitis development was next assessed, where 1 × 10^6^ LPS-polarized BMDMs from Gab2/3^−/−^ or WT were adoptively transferred into Rag2^−/−^ mice first, and then sorted naïve WT CD4^+^ T-cells were transferred 24 h later. From two independent experiments, weight loss was greater for the naïve CD4^+^ T-cell transferred recipient mice treated with Gab2/3^−/−^ BMDMs compared to the naïve CD4^+^ T-cell transferred recipient mice treated with WT BMDMs (Figure 5D). At 2–5 weeks post-transfer, the Gab2/3^−/−^ BMDM group had a significantly larger body weight loss when compared to the WT BMDM group at each time point. This indicates that Gab2/3^−/−^ BMDMs were more potent at colitis induction than WT BMDMs. Although the colon weight/length ratio did not reach statistical significance, there was a trend (Figure S9).

### Gab2/3^−/−^ CD8^+^ T-Cells Promote Colitis Following Adoptive Transfer into Immune-Deficient Hosts

**Figure 6:**
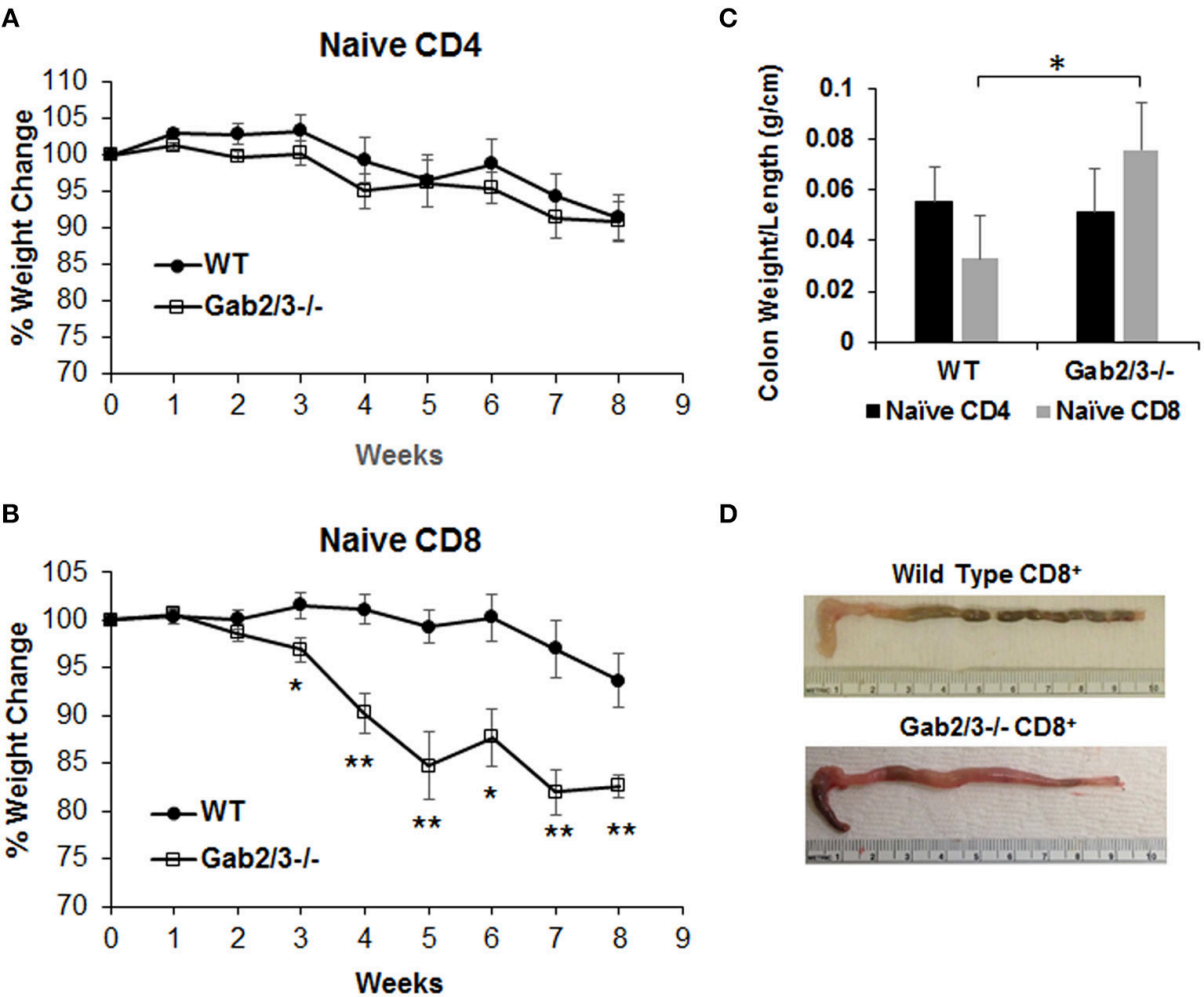
Adoptively transferred naïve Gab2/3^−/−^ CD8^+^ T-cells are more colitogenic than WT CD8^+^ T-cells. **(A)** FACS sorted naïve CD4^+^CD45RB^high^ cells from WT or Gab2/3^−/−^ mice were transferred into Rag2^−/−^ mice via IP injection (8 × 10^5^ cells/recipient). The weight change was monitored weekly. The results shown are combined from two independent experiments and are shown as the mean ± SE, for *N* = 12 from WT naïve CD4 and *N* = 16 for Gab2/3^−/−^ naïve CD4. There was no significant difference between WT and Gab2/3^−/−^ naïve CD4^+^ cell-transferred mice (unpaired *t*-test). **(B)** FACS isolated naïve CD8^+^ cells (CD8^+^CD44^−^CD62L^+^) from WT or Gab2/3^−/−^ mice were transferred into Rag2^−/−^ mice via IP injection (4 × 10^5^ cells/recipient). The weight change was monitored weekly. The results shown are combined from 2 independent experiments and are shown as the mean ± SE, for *N* = 10 for WT and Gab2/3^−/−^ naïve CD8 (^*^P < 0.05, ^**^P < 0.01, unpaired *t*-test). **(C)** The ratio of colon weight/length is shown. Upon euthanasia, the colon weight and length were measured (data are presented as the mean ± SD, *N* = 8–9 mice per group, ^*^P < 0.05; unpaired *t*-test). **(D)** Representative examples of the colon comparing WT to Gab2/3^−/−^ naïve CD8^+^ T-cell adoptive transfer recipients.

To test T-cell function *in vivo*, either naïve CD4^+^ T-cells (CD4^+^CD45RB^high^) or naïve CD8^+^ T-cells (CD8^+^CD44^−^CD62L^+^) were sorted from WT or Gab2/3^−/−^ mice. Representative plots of T-cell flow cytometry gating and intracellular staining are shown in Figure S10. Upon transferring sorted naïve T-cells (Figure S11) into Rag2^−/−^ mice by IP injection, both induced colitis as expected from the prior lack of antigen exposure. Weight loss for the transferred recipients was not different for transferred naïve Gab2/3^−/−^ CD4^+^ T-cells relative to WT (Figure 6A), but was greater for naïve CD8^+^ T-cells from Gab2/3^−/−^ compared to WT (Figure 6B) (Figure S12). At 3–8 weeks post-transfer, the Gab2/3^−/−^ group had significantly larger weight loss compared to the WT group at each time point and half of the Gab2/3^−/−^ group mice required euthanization due to more than 25% body weight reduction. The colon weight/length ratio was significantly increased in mice transferred with naïve Gab2/3^−/−^ CD8^+^ T-cells compared to WT but not for CD4^+^ T-cells (Figure 6C). A representative gross colon comparison is shown in Figure 6D and illustrates the thickening, shortened length, and loss of normal stool consistency compared with WT. Histology revealed extensive colitis in both groups at the time of euthanasia according to defined weight loss criteria (≤75% normal body weight) or at the termination of the experiment (week 8).

### Increased Numbers of Activated Gab2/3^−/−^ CD8^+^ Intraepithelial Effector Cells in the Colon

**Figure 7:**
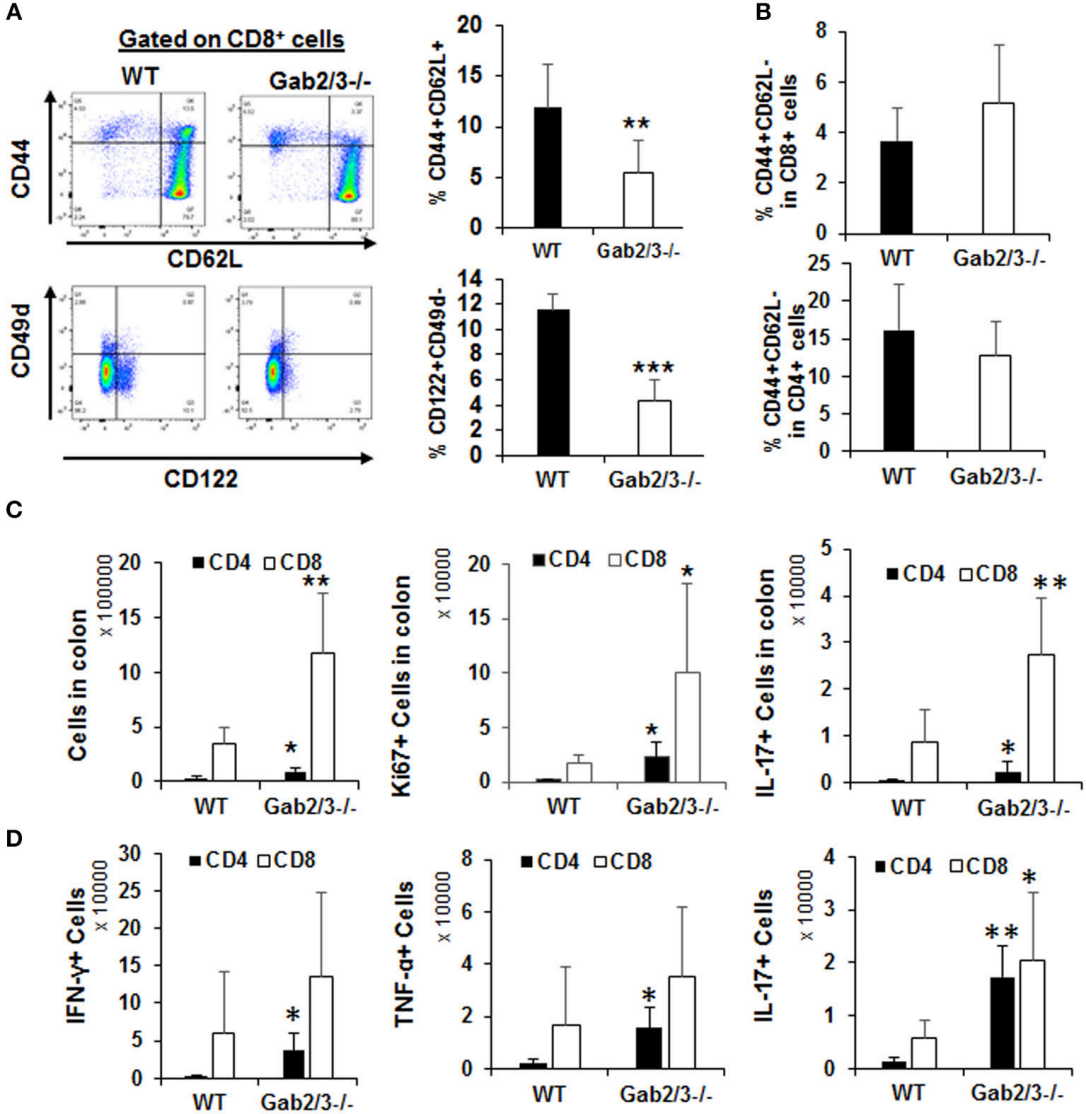
Gab2/3^−/−^ CD8^+^ intraepithelial T-cells have greater *in vivo* effector activities. **(A)** Freshly isolated splenocytes from WT and Gab2/3^−/−^ mice were stained with CD4, CD8, CD44, CD62L, CD122, CD49d, and Sca1 antibodies. A representative histogram and gating strategy is shown on the left. Gating on CD8^+^ cells, the percentage of CD44^+^CD62L^+^ and CD122^+^CD49d^−^ is shown as the mean ± SD, *N* = 6, ^**^P < 0.01; ^***^P < 0.001; unpaired *t*-test). Gab2/3^−/−^ mouse spleen had a reduced population of CD44^+^CD62L^+^ and CD122^+^CD49d^−^ cells. **(B)** No change in either CD4^+^ or CD8^+^ splenocytes with the CD44^+^CD62L^−^ effector phenotype. **(C)** The total number of intraepithelial lymphocytes, Ki67^+^, and IL-17^+^ cells in colons from WT and Gab2/3^−/−^ mice (For total and Ki67, *N* = 6; For IL-17, *N* = 6–7; ^**^P < 0.01; ^*^P < 0.05; unpaired *t*-test). **(D**) IELs were isolated and stimulated for 4 h with 50 ng/ml PMA/750 ng/ml ionomycin/5μg/ml BFA and the levels of intracellular IFNγ, TNFα, and IL-17 were measured by flow cytometry (*N* = 5; ^**^P < 0.01; ^*^P < 0.05; unpaired *t*-test). All data are shown as the mean ± SD.

In addition to the regulatory and effector phenotypes, a balance between effector and memory phenotype characterizes the immune response. The memory phenotype is characterized by high levels of expression of the activation marker CD44 and loss of CD62L. Virtual memory or memory phenotype cells are IL-15 dependent and mediate antigen non-specific bystander protective functions (48). Virtual memory cells decrease with age, leading to effector T-cell immunosenescence, changes in gut microbiota, and development of IBD in humans when exposed to antigens in the gut. Therefore, the immunophenotypes of Gab2/3^−/−^ T-cells were examined. Although there were no changes in the percentage of CD4^+^ and CD8^+^ T-cells between WT and Gab2/3^−/−^ mouse spleen, a highly consistent reduction of both the CD8^+^CD44^+^CD62L^+^ and the CD8^+^CD122^+^CD49d^−^ sub-populations was observed in Gab2/3^−/−^ mice relative to WT, resulting in loss of memory phenotype T-cells which include regulatory CD8^+^ T-cell populations (CD44^+^CD62L^+^CD122^+^) (Figure 7A). The CD44^+^CD62L^−^ effector population of both splenic CD4^+^ and CD8^+^ T-cells was not changed (Figure 7B). Since T-cells can have both regulatory or effector functions and based on known markers for these subsets of T-cells (49–51), flow cytometry analyses were performed. Regulatory T-cells (Treg) are major suppressors of T-cell activation that are characterized by expression of CD25 and FoxP3 (52). To determine whether Gab2/3 control CD4^+^ Treg development, Tregs were quantified by flow cytometry and assessed for functional suppressor activity. The CD4^+^CD25^+^FoxP3^+^ population was not significantly changed and normal suppressor cell activity was maintained *in vitro* (Figures S13A,B). CD4^+^ T-cell subsets were analyzed further for Th1, Th2, and Th17 differentiation in culture (Figures S13C-E) which are characterized by differential expression of intracellular cytokines. There were no significant changes in CD4^+^ T-cell development into Th1, Th2, and Th17.

T-cells are key cells of the adaptive immune system and have important effector functions in colitis through production of IFNγ. To determine whether gut T-cells were also more activated at the site of antigen presentation, cells were isolated from either mesenteric lymph nodes (MLN) or large intestine including the intraepithelial lymphocytes (IEL) and lamina propria lymphocytes (LPL). IEL and LPL fractions were isolated using a commercially available kit (Miltenyi Biotec) and it was important to analyze these cells since they are the tissue-resident lymphocytes acutely responsible for antigen-dependent responses in the gut and for maintaining colon immune homeostasis. All T-cell subsets were analyzed by flow cytometry immunophenotyping and cell cycle analysis using Ki67 staining. There was no significant difference regarding either the number of T-cells or their proliferation status in the LPL or MLN (Figure S14). However, strikingly there was significantly increased numbers of CD8^+^ T-cells in the IEL fraction relative to WT and these cells were increasingly proliferative (Ki67^+^) and expressed IL-17 (Figure 7C). Following stimulation with PMA/ionomycin *in vitro*, expression of IFNγ and TNFα was detected in both CD4^+^ and CD8^+^ IELs (Figure 7D). Despite the higher induction of these cytokines in CD4^+^ IELs, the CD8^+^IFNγ^+^ and CD8^+^TNFα^+^ fractions were more abundant relative to CD4^+^IFNγ+ and CD4^+^ TNFα^+^ fractions. Importantly, cytokine receptor expression levels were determined for IL-2Rβ and the common γ chain and following IL-2 stimulation. No significant changes were observed, thus defective cytokine response was not due to altered receptor expression but rather altered signaling downstream of the receptor (data not shown).

### Dysregulated PI3K/Akt/mTOR Pathway Signaling in Gab2/3^−/−^ Macrophages and T-Cells

Because adaptor proteins function directly at the level of protein-protein interactions and phosphorylation, to understand the molecular mechanism of signaling dysregulation in Gab2/3^−/−^ T-cells and macrophages, isolated primary cultured splenic T-cells and BMDMs were utilized for Western blot analysis. Signaling pathways known to be modulated by Gab adaptor proteins were examined with a central focus on the PI3K/Akt/mTOR pathway (53, 54).

**Figure 8:**
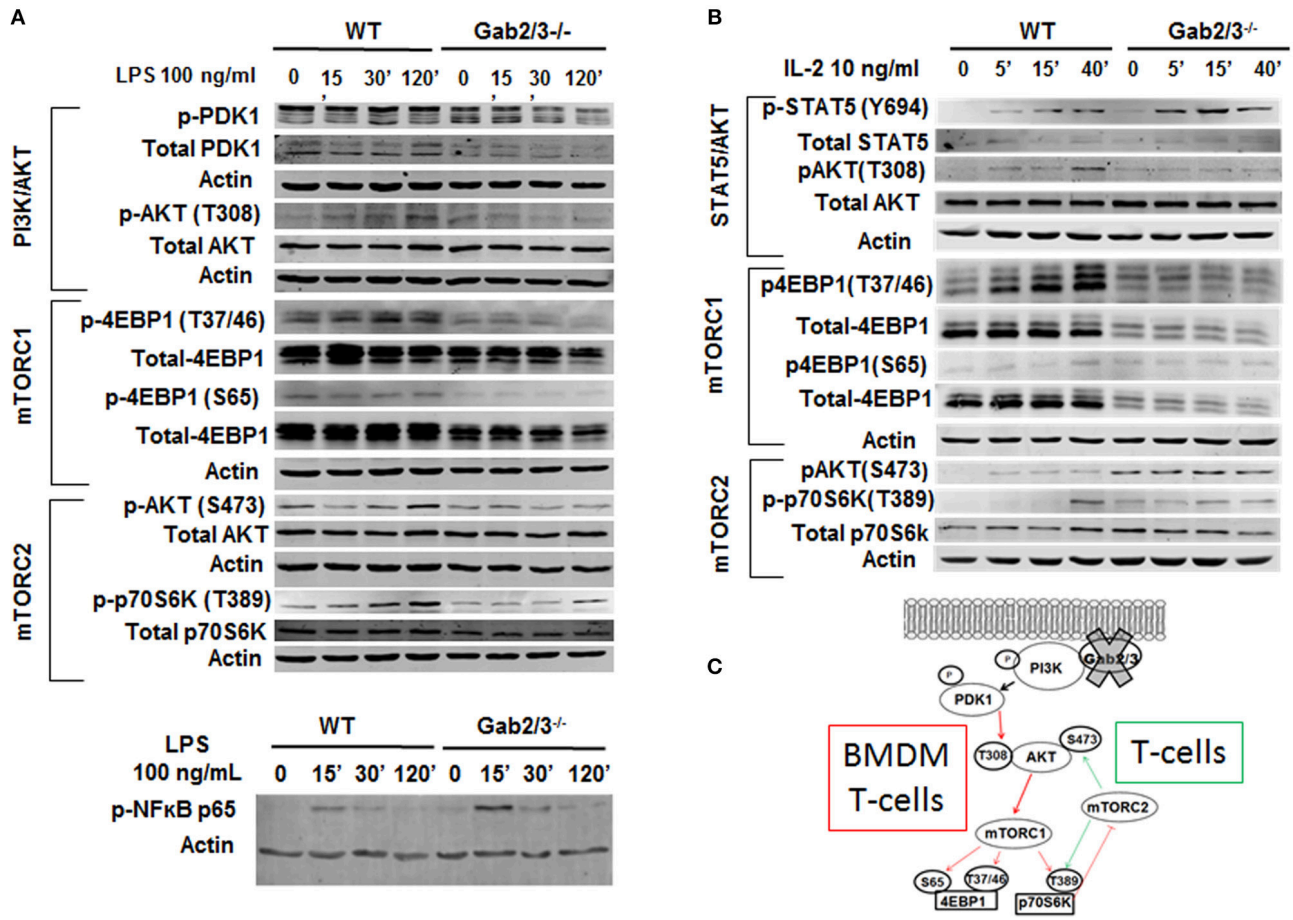
Dysregulated PI3K/AKT/mTOR signaling in Gab2/3^−/−^ BMDMs and T-cells. **(A)** Mature BMDMs were analyzed for the protein expression of several intermediates of the PI3K/AKT/mTOR signaling pathway following 0, 30, and 120 min of stimulation with 100 ng/ml LPS. Changes in PI3K/AKT/mTOR signaling pathway components including AKT, p70S6K, and 4EBP1 were observed and representative blots are shown. **(B)** Splenic T-cells were analyzed by Western blot for STAT5 and signaling molecules in the PI3K/AKT/mTORC1 pathway. WT and Gab2/3^−/−^ T-cells were treated with or without 10 ng/mL IL-2. Representative blots are shown. **(C)** Schematic illustrations of roles for Gab2/3 in promoting PI3K/Akt/mTOR pathway activation with key phosphorylation sites shown. Inhibited pathways involving mTORC1 are shown with thin red arrows resulting in loss of negative feedback regulation in activated T-cells but not activated macrophages. T-cell specific gain of mTORC2 is reflected by the green arrows which activate AKT by phosphorylation of S473.

BMDMs were treated with 100 ng/ml LPS for up to 120 min, and Gab2/3 deletion reduced phosphorylated PDK1 and phosphorylated AKT at the site of T308 relative to WT. Gab2/3 deletion also severely reduced the amount of phosphorylated 4EBP1 (Ser65 and Thr37/46) (Figure 8A) compared to WT. Gab2/3^−/−^ BMDMs also showed elevated p-NFκB p65 (Ser536) above that of WT BMDMs at the same time points. The T-cell response to IL-2 was initially marked by STAT5 phosphorylation in the double knockout splenic T-cells by Western analysis in addition to dysregulated PI3K/Akt/mTOR pathway (Figure 8B). In BMDMs, loss of mTORC1 did not increase mTORC2 activity. However, in T-cells the levels of p70S6K T389 and Akt S473 phosphorylation were increased relative to WT due to Gab2/3 deletion (green arrows). These results suggest that increases in phosphorylation of p70S6K and Akt (S473) are due to mTORC2 consistent with a p70S6K-mediated loss of the mTORC1-mTORC2 negative feedback regulation in T-cells (55) (Figure 8C). Quantitation of the representative Western blot data is shown in Figure S15.

## Discussion

In this study redundancy among Gab2 and Gab3 was uncovered through generation of novel double knockout mice. The results revealed previously unappreciated negative regulatory roles in prevention of spontaneous colitis and suppression of DSS-induced colitis. Mechanistic studies further defined that the cause of colitis was not due to deletion of Gab2/3 in colon epithelium but rather due to dysregulated immune cell biology. These findings provide greater understanding of the specific cell types that require Gab-mediated signaling and highlight how signaling dysregulation in naïve CD8^+^ T-cells and macrophages cause colitis development. These insights might also lead to new treatment approaches in the future.

Previous studies have shown that in overexpression systems Gab2 plays a role in T-cell receptor (TCR) signaling in Jurkat cells and mediates a feedback inhibitory PI3K signal during TCR activation (31, 32, 34). A Gab2 proteomic study performed in Jurkat T-cells, confirmed that Gab2 interacts with p85 and the 3 catalytic PI3K p110 subunits (α, β, δ) (56). However, these prior studies were mainly performed in cell lines and focused on TCR signaling. One study of T-cell responses from 129 × C57BL/6 mixed background mice showed increased responses to anti-CD3/CD28 in Gab2^−/−^ primary T-cells (33). However, in the studies reported here using extensively backcrossed C57BL/6 mice, we did not observe effects of Gab2 or Gab2/3 deficiency on CD4^+^, or CD8^+^ T-cell proliferation upon TCR activation *in vitro*, or CD4^+^ T-cell differentiation into Th1, Th2, or Th17 lineages *in vitro*. It is important to study Gab2/3 signaling in these cells since they are major regulators of normal colon health. Gab2 is phosphorylated following IL-2 and IL-15 stimulation of Jurkat cells, however a functional role in IL-2 signaling has not been established. These findings provide new information for the field and suggest new avenues for therapy by restoring Gab adaptor protein activity.

In T-cells, Gab2 is induced downstream of IL-2 and IL-15 signaling (35). We found that Gab2/3^−/−^ T-cell phenotypes are most similar to the p110δ knockout mice that develop colitis. Although negative regulation of T-cell activation by Gab2 has already been described through structure-function studies in Jurkat T-cells, no *in vivo* evidence of pathology has been reported and there is little data generated in primary lymphocytes. Our data indicate that Gab2 deficiency alone is insufficient and that additional loss of compensatory functions by Gab3 is required to release T-cells from Gab-mediated repression sufficiently to cause pathology. CD8^+^ T-cell profiling predicts prognosis in patients with Crohn's disease and ulcerative colitis (5, 57) and we observed the main contribution to colitis development was mediated by cytokine producing, proliferative, CD8^+^ Gab2/3^−/−^ T-cells in the intraepithelial compartment. In contrast, CD4^+^ T-cells had a smaller numerical contribution. This specificity might derive from the fact that the highest Gab3 expression occurs in memory CD8^+^ T-cells and intraepithelial lymphocytes, although detailed analysis of the mucosal IEL sub-types involved will be a subject of future study. It is possible that Gab2 is a key negative regulator of the effector activity whereas Gab3 plays a complementary role in promoting memory phenotype. With these dual roles, combined deletion would be expected to yield synergistic effects. Gab2/3 function in IL-15 signaling was observed (data not shown) which may account for the reduced memory phenotype. Future studies of memory function in mice lacking Gab2/3 are warranted. Interestingly, given the important role for IL-17/IL-23 signaling in colitis (58, 59), the observed increase in Tc17 cells lacking Gab2/3 provides new insights toward not only Gab2/3 regulation downstream of cytokines but also in regulation of important cytokine producing cells.

In macrophages, the absence of Gab2 and Gab3 led to a heightened response following TLR stimulation. The BMDMs were more pro-inflammatory, with high levels of IFNγ and TNFα protein. A direct role for Gab2/3 downstream of TLR signaling has not been described but identification of a role for Gab2/3 downstream of TLR activation is novel. Inhibition of PI3K also leads to enhanced LPS-mediated activation of JNK, p38, and ERK1/2 pathway in monocytes (60) therefore this was a major focus. This same study showed increased NF-κB pathway upon PI3K p110γ inhibition, consistent with our observation which shows increased NF-κB pathway. However, NF-κB activation has only been reported with knockout of PI3K class IB PI3K p101/p110γ, but Gab deficiency acts primarily via class IA PI3K p85/p110α, β, γ. Notably, we found that deletion of Gab2/3 in BMDMs reduced mTORC1 activation but without compensatory increased mTORC2 which can promote NF-κB activation (61, 62) in some cell types. mTOR activation is associated with macrophage polarization (63) and PI3K p110γ knockout/inhibition reprograms macrophages to an M1 phenotype (12) whereas PI3K p110δ knockout reduces macrophage IL-10 production (8, 10). A positive role for Gab1/2 in M2 macrophage polarization and idiopathic pulmonary fibrosis has been described (64). Similarly, mTORC2 promotes M2 polarization (9, 65). In the BMDM adoptive transfer experiments, Gab2/3^−/−^ BMDMs promoted inflammation relative to WT.

There are several common traits in Gab2/3^−/−^ T-cells and macrophages with respect to the PI3K pathway. Both cell types showed the same reduction in PI3K/AKT^T308^/mTORC1/4EBP1 (marked by the red arrows in Figure 8C). Decreased mTORC1 and increased pSTAT5 is unique from other existing mouse models of PI3K, AKT, or mTOR deletion. Consistent with the observed mTOR dysregulation, T-stem cell memory (Tscm) accumulation and Sca1 expression (data not shown) were consistent with reports of Raptor inhibition (17). In contrast, Rictor inhibits memory T-cells (20) and thus gain of mTORC2 activity would suppress memory phenotype cooperatively with decreased IL-15 responsiveness. Both Rictor and Raptor inhibition (18) increase memory T-cells because they are normally critical for glycolysis needed to drive effector cell function (19). PI3K p110δ deletion results in inactivation of CD4^+^ Tregs (66). Despite CD4^+^ regulatory T-cells being major suppressors of immune cell activation, they were not reduced in the Gab2/3^−/−^ mice. Therefore, the transplantable disease does not appear to be mediated through dysregulated regulatory CD4^+^ T-cell number or function. This difference between the role of Gab expression compared with that of deletion of the p85-interacting subunits could be due to the concomitant increased activation of STAT5, a known regulator of CD4^+^ Treg development through Foxp3 expression. However, reduced CD8^+^ Treg numbers was observed although the phenotypic [PD1^+^CD8^+^ fraction (50, 67)] and functional significance has not yet been studied. Both *in vitro* and *in vivo* CD8^+^ T-cell characterization revealed a major role for Gab2/3 downstream of the IL-2Rβ (CD122) for coordinating IL-2/IL-15 (35) but not IL-7 signaling. In addition to modulation of CD122 function (68), gain of pSTAT5 could also be an important contributor that drives effector proliferation (69) at the site of infection to sustain chronic colitis.

It is also interesting that we observed early invasive cancer in some of the transplant recipients but not in the knockout mice themselves. A major difference between these models is that the epithelial cells are WT in the BM transplant model and thus transformation of the epithelium may be more susceptible than epithelium lacking Gab2/3. This remains to be directly tested and future studies will be needed to examine roles for Gabs in epithelial barrier function or in transformation. Alternatively, irradiation itself could necessitate epithelial repair and proliferation that is essential for transformation or could induce genetic damage leading to mutations that drive epithelial transformation. In contrast, epithelial-specific knockout of SHP-2, a Gab2 partner protein, causes barrier defects and colitis (70). Gab2 overexpression is also a clinically significant biomarker for malignant invasion and metastasis of several cancers, including breast and gastric cancer (71–75). It is possible that loss of Gab-SHP-2 interaction could play a role in our model. We show in this study a major role for Gabs in mTORC1 activation in macrophages and T-cells, and mTORC1 is a major gastrointestinal cancer promoting signal in epithelium (76). Therefore, dual roles for Gabs in mTORC1 activation in different tissues are possibly and could account for some of the differences we have observed between transplanted and non-transplanted mice in regard to epithelial transformation.

These studies did not examine the role of Gab1 in immune cell function. However, we did find that Gab1 levels are increased in Gab2/3^−/−^ macrophages (data not shown) which suggests that Gab1 might be part of a compensatory response to moderate the defects and could contribute to some mouse-to-mouse variation in addition to cage-specific microbiota. Gab1 is also known to promote TLR3/4 and RIG-I mediated production of pro-inflammatory cytokines and type I interferon in primary macrophages and mouse embryonic fibroblasts (77) and to promote mTORC2 activation (78). Future studies that target Gab1 in combination with Gab2/3 may shed light on the combined role of all Gabs in regulation of immune cell activation and colitis development.

It is possible that human IBD involves changes in Gab family member expression within the T-cells and/or macrophages but a role for Gab dosage as a human IBD biomarker remains to be determined. Human CD8^+^ T-cells are emerging as major mediators of human IBD and the identification of a key role for Gab2/3 in suppression of T-cell and macrophage function, fills in major gaps in our understanding of how CD8^+^ T-cells drive IBD. Furthermore, the H. pylori virulence factor CagA has been shown to protect against DSS-induced experimental colitis (79) and is inversely associated with human IBD (40) but no functional roles have been previously attributed to Gab adaptor proteins in promoting a healthy colon under normal physiological conditions. The findings of this study thus provide a deeper understanding of how Gab2 and Gab3 redundantly function to ultimately control CD8^+^ T-cell and this finding could lead to new therapeutic avenues.

## Data Availability

All datasets generated for this study are included in the manuscript and/or the supplementary files.

## Author Contributions

ZW, TV, WZ, YC, MM, GF, and HN performed experiments and analyzed the data. TV and SB performed pathology analyses. PH-G backcrossed Gab3^−/−^ mice to C57BL/6 and provided them for this study. AN and CP provided advice and performed DSS experiments in their laboratory (HN). DW and RW provided advice and performed CD4^+^ T-cell experiments in their laboratory (YC and GF). ZW, TV, RW, and KB wrote the manuscript. KB organized the study and performed colitis, macrophage, and CD8^+^ T-cell experiments in his laboratory (ZW, TV, WZ, and MM).

### Conflict of Interest Statement

KB has a financial interest in Valhalla Scientific Editing Service, LLC. The remaining authors declare that the research was conducted in the absence of any commercial or financial relationships that could be construed as a potential conflict of interest.
