# Supplementary material for: Gab2 and Gab3 Redundantly Suppress Colitis by Modulating Macrophage and CD8+ T-Cell Activation
Source: Front Immunol. 2019 Mar 18;10:486. doi: 10.3389/fimmu.2019.00486 (PMC6431666; doi:10.3389/fimmu.2019.00486)
Supplement: Supplementary file 1 [file Data_Sheet_1.docx]

**Supplemental Materials and Methods, Figures, and Figure Legends**

Supplemental Materials and Methods

*Flow cytometry to check the purity of BMDM cells -* After 6 days of culture, BMDM cells were removed from the plates with ice-cold PBS. The cells were first pre-incubated with purified anti-CD16/CD32 antibody (2.4G2) for 5-10 minutes on ice, then stained with either FITC-F4/80 antibody plus APC-CD11b antibody or with isotype controls (Rat IgG2a, κ Isotype Control, FITC and Rat IgG2b, κ Isotype Control, APC) for 30 minutes at 4 °C. The antibodies used are listed in Table S1. Data were acquired with a BD LSRII (BD Biosciences, San Jose, CA) and analyzed with FlowJo Software (FlowJo, LLC. Ashland, OR).

*FACS sorting of naïve CD4^+^ (CD4^+^CD45RB ^high^) and naïve CD8^+^ (CD8^+^CD44^-^CD62L^+^) T-cells for adoptive transfer experiments.* Splenic T-cells were isolated from either wild-type or Gab2/3^-/-^ spleen and then filtered through a 70 µM strainer into a 50 mL conical tube and cells were centrifuged at 450 x g for 7 minutes. The cells were gently resuspended in 5 mL per spleen of red cell lysis solution (155 mM NH_4_Cl, 12 mM NaHCO_3_, 0.1 mM EDTA) for 10 minutes at room temperature. After lysis of red cells, enrichment of CD4^+^ or CD8^+^ T cells was carried out using CD4 (L3T4) mouse MicroBeads or CD8a (Ly-2) mouse MicroBeads from Miltenyi Biotec (Auburn CA) following the manufacture user’s manual. Enriched CD4^+^ cells were first blocked by pre-incubation with purified anti-CD16/CD32 antibody (2.4G2) for 5-10 minutes on ice, and then stained with FITC-CD4 monoclonal antibody and PE-CD45RB antibody or an aliquot stained with isotype controls (Rat IgG2b, κ Isotype control, FITC, and Rat IgG2a, κ Isotype control, PE) and incubated on ice protected from light for 30 minutes. For enriched CD8^+^ cells, similar pre-incubation with purified anti-CD16/CD32 antibody (2.4G2) for 5-10 minutes on ice, followed by staining with FITC-CD8a monoclonal antibody, PE-CD62L (L-selectin) monoclonal antibody, and APC-CD44 monoclonal antibody was done. An aliquot was also stained with isotype controls (Rat IgG2b, κ Isotype control, FITC, and Rat IgG2a, κ Isotype control, PE, and Rat IgG2b, κ Isotype Control, APC) and incubated on ice protected from light for 30 minutes. Antibody details are listed in **Table S1**. All labelled cells were washed, resuspended, and passed through a 70 µM strainer into a FACS tube. Cells were kept on ice protected from light until sorting. Naïve CD4^+^ or naïve CD8^+^ cells were sorted using a Sony SH800 Cell sorter (Sony sorting chip nozzle size: 100 micron; selected lasers: 488 nm, 561 nm and 638 nm; filter setting: FL2 525/50, FL3 600/60, FL4 665/30; sorting mode: purity). Initial instrument setup was done according to the Sony SH800 guidelines and on screen instructions. Auto calibration was performed using Sony automatic setup beads. Set up and appropriate compensation were determined on the cell sorter with unstained cells and single-stained controls. Set up gating for naïve CD4^+^ or naïve CD8^+^ cells. Cells were sorted into 15 mL collection tube containing 1.5 mL fetal bovine serum (5 ºC). An aliquot of each sorted cell population was run to assess purity (>97%). The sorting yield was ~50% depending on the sorting speed.

*Serum analysis on mice with rectal prolapse -* Mouse blood was collected by retro-orbital bleeding and samples sit at room temperature for 15-20 minutes. Serum was separated by centrifugation at 1000 x g for 10 minutes in a refrigerated centrifuge. The serum was sent to the Quality Assurance & Diagnostic Lab, Division of Animal Resources at Emory University on the day of serum collection for the Comprehensive Diagnostic Profile (Cat # 500-0038, Patterson Veterinary, Saint Paul, MN).

*Endoscopy of live DSS-treated mice and histology on euthanized mice -* A high-resolution colonoscopy system (Becker et al., 2005) was used to monitor mouse colons at day 0, 7, and 14. This system consisted of a miniature rigid endoscope (1.9-mm outer diameter), a xenon light source, and a triple-chip high-resolution CCD camera. Endoscopic procedures were viewed with high-resolution (1,024 × 768 pixels) images on a flat-panel color monitor.

*T-cell CD4^+^ in vitro differentiation and Treg suppressor activity assay* – Four major CD4^+^ T-cell subsets, Treg, Th1, Th2, and Th17 cells were differentiated from naïve CD4^+^ T-cells upon ligation of their T-cell receptors with antigens, depending on the cytokines they received as described (Sekiya and Yoshimura, 2016). To assess Treg suppressor activity, naive CD4^+^ T-cells (CD4^+^CD25^−^, 2 × 10^4^) pooled from spleen and lymph node were stimulated with 2 µg/ml α-CD3 in the presence of 8 × 10^4^ irradiated (3000 rads) splenocytes in 200 µl culture medium in a round-bottomed plate. Indicated portions of FACS-sorted CD4^+^CD25^+^ Treg cells from the spleen and lymph nodes were added at the beginning of the culture. After 48 hours of culture, the cells were pulsed and harvested as previously described (Zeng et al., 2008).

Supplemental Figures and legends

**
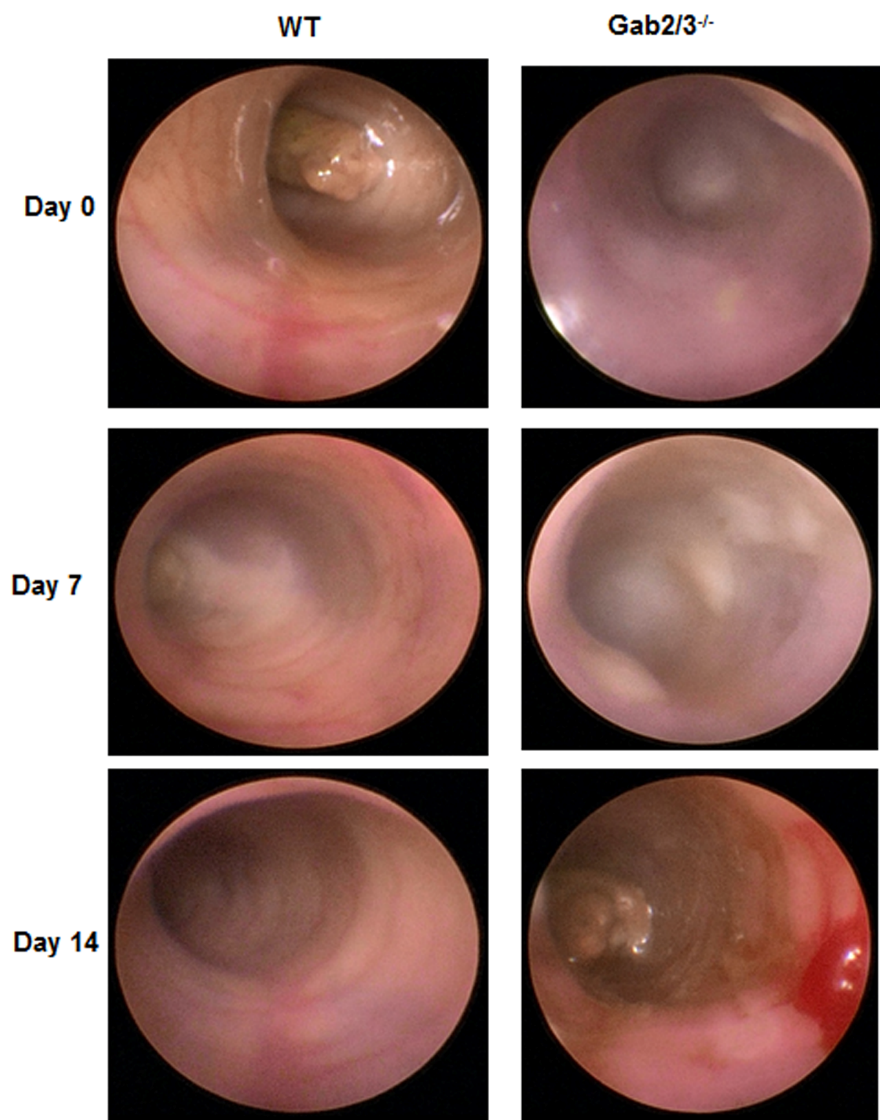
**

**Figure S1. Representative endoscopic image from WT and Gab2/3^-/-^ mouse colon**. WT and Gab2/3^-/-^ mice were assessed following 2.5% DSS treatment. Mice were analyzed by endoscopy at Days 0, 7, and 14. Images shown were captured from videos generated during imaging analysis.

**
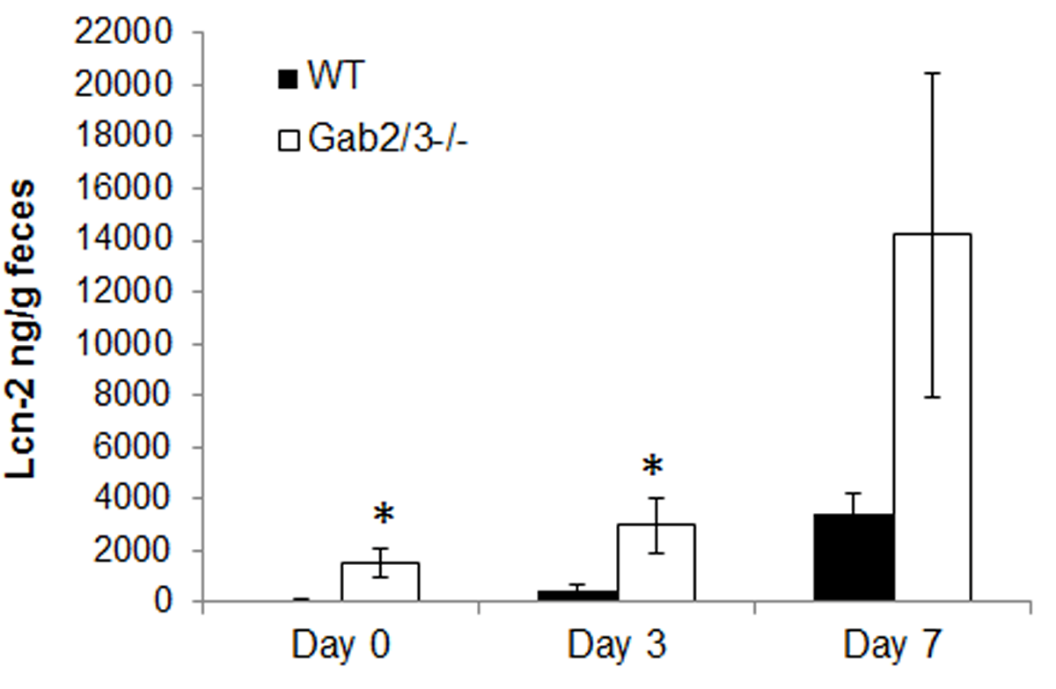
**

**Figure S2. DSS-induced colitis increases lipocalin-2 (Lcn-2) levels in Gab2/3^-/-^ mice compared to WT.** WT and Gab2/3^-/-^ mice were assessed following 2.5% DSS treatment. Stool samples were collected from mice at Days 0, 3, and 7 and Lcn-2 enzyme was analyzed by ELISA based assay. WT, N=14; Gab2/3^-/-^, N=9-11; *P<0.05, unpaired t-test.


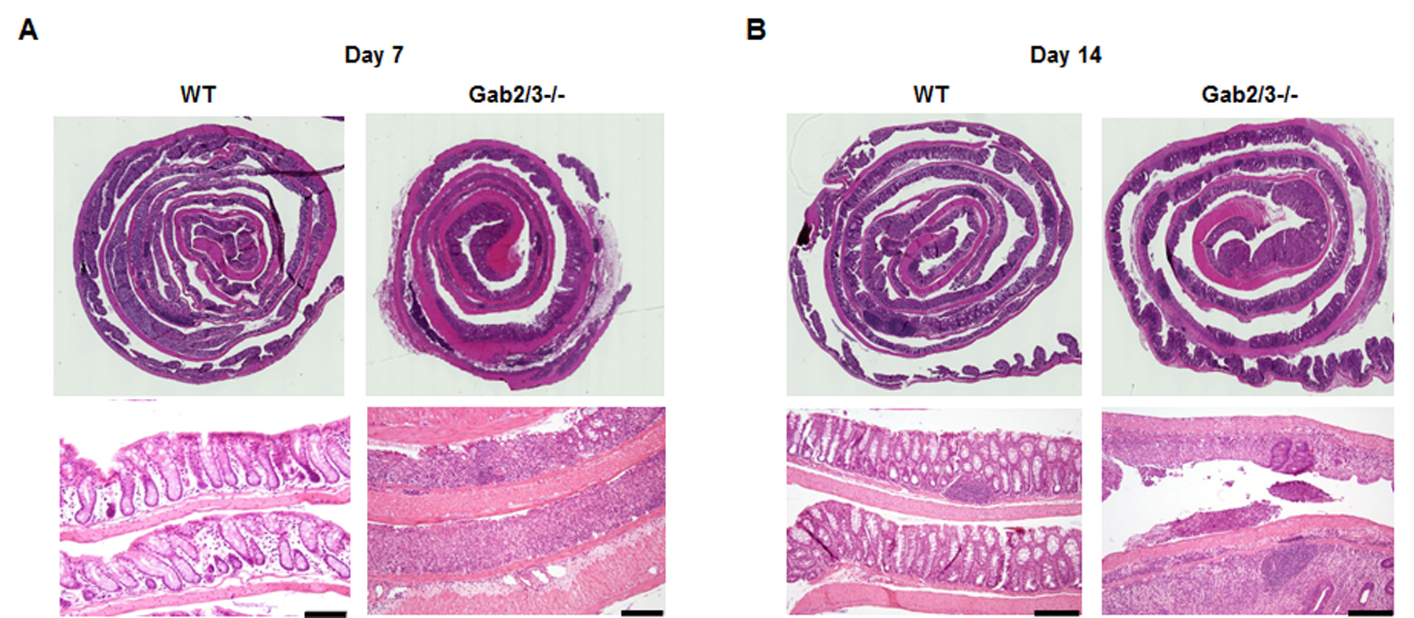


**Figure S3. Histological analysis of colitis in DSS-treated mice.** WT and Gab2/3^-/-^ mice were assessed following 2.5% DSS treatment. **a)** Histology of Swiss roll mounts and colon histology of DSS-treated WT and Gab2/3^-/-^ mice at day 7. **b)** Histology of Swiss roll mounts and colon histology of DSS-treated WT and Gab2/3^-/-^ mice at day 14.


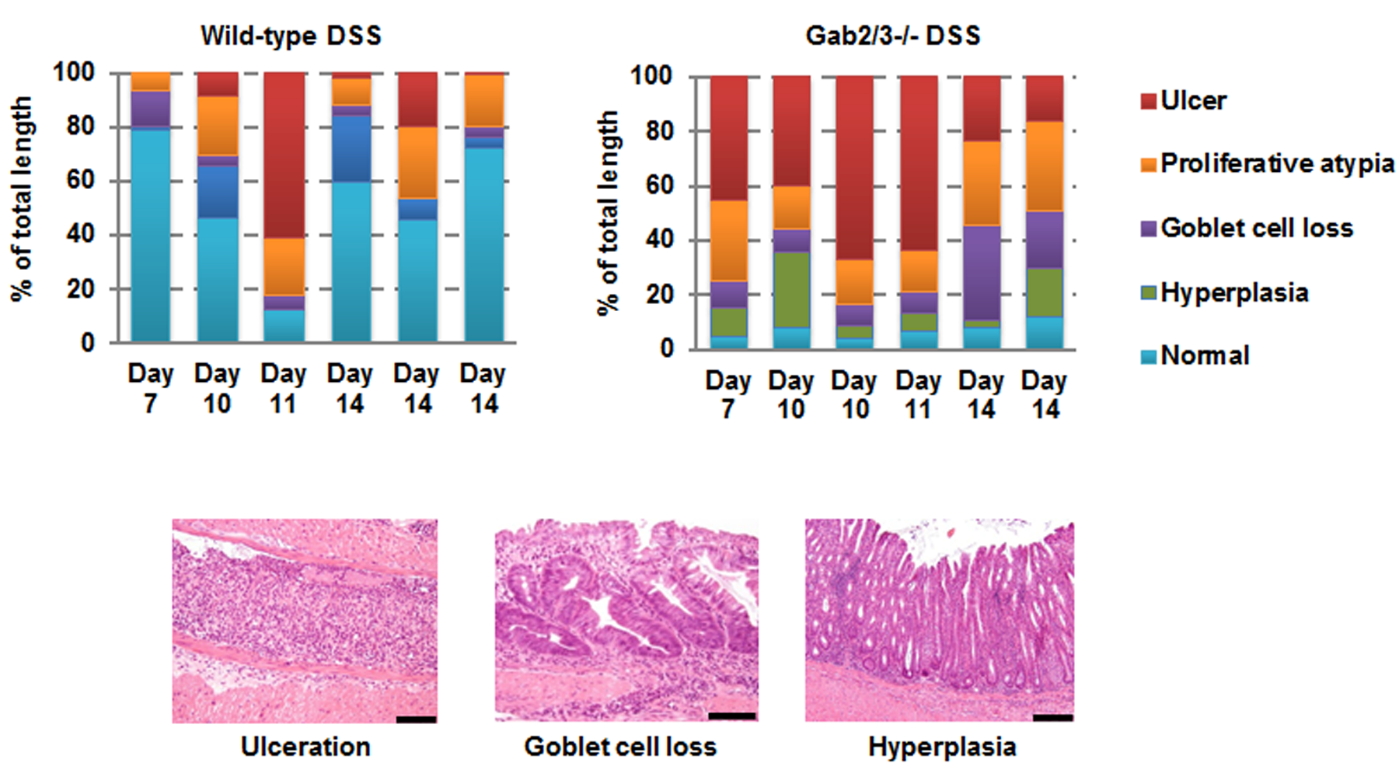


**Figure S4. Scoring criteria and percentages of diseased colon in DSS-treated WT and Gab2/3^-/-^ mice.** WT and Gab2/3^-/-^ mice were assessed following 2.5% DSS treatment. Histology slides from euthanized mouse colon were assessed for the average percentage of hyperplasia, proliferative atypia, loss of crypts, and goblet cells as described in detail in the Materials and Methods. Percentages were calculated based on the full length of the colon. An example of colon histology with ulceration, goblet cell loss, or hyperplasia is shown.


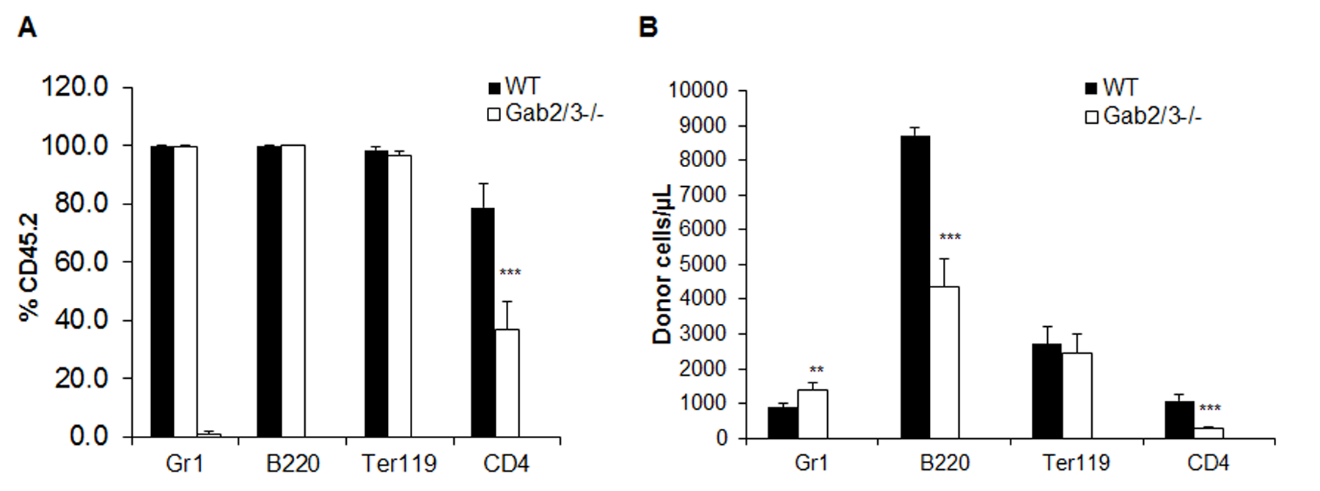


**Figure S5: Donor cell engraftment in the peripheral blood of WT or Gab2/3^-/-^ bone marrow transplanted mice.** Bone marrow isolated from WT or Gab2/3^-/-^ mice (CD45.2) was intravenously injected into lethally irradiated WT BoyJ mice (CD45.1) at 1 x 10^7^ BM cells/recipient. Experiments were repeated twice (N=10 mice total for each group) and the results shown are the mean ± SD, **P<0.01, ***P<0.001, unpaired t-test. **a)**. Percentage of donor CD45.2 engraftment in Gr1, B220, Ter119, and CD4 positive cells in the peripheral blood of wild-type or Gab2/3^-/-^ bone marrow transplanted mice 8 weeks following transplantation. **b)**. Donor-derived cells in the peripheral blood of wild-type or Gab2/3^-/-^ bone marrow transplanted mice 8 weeks following transplantation. Total white blood cell (WBC) counts of wild-type or Gab2/3^-/-^ bone marrow transplanted mice were determined using a HemaTrue Hematology Analyzer (Heska, Loveland, Colorado). The percentage of Gr1, B220, Ter119, CD4 positive donor cells in the peripheral blood was determined by staining lysed peripheral blood cells with Gr1, B220, Ter119, or CD4 antibodies along with antibody to CD45.2 positive cells.


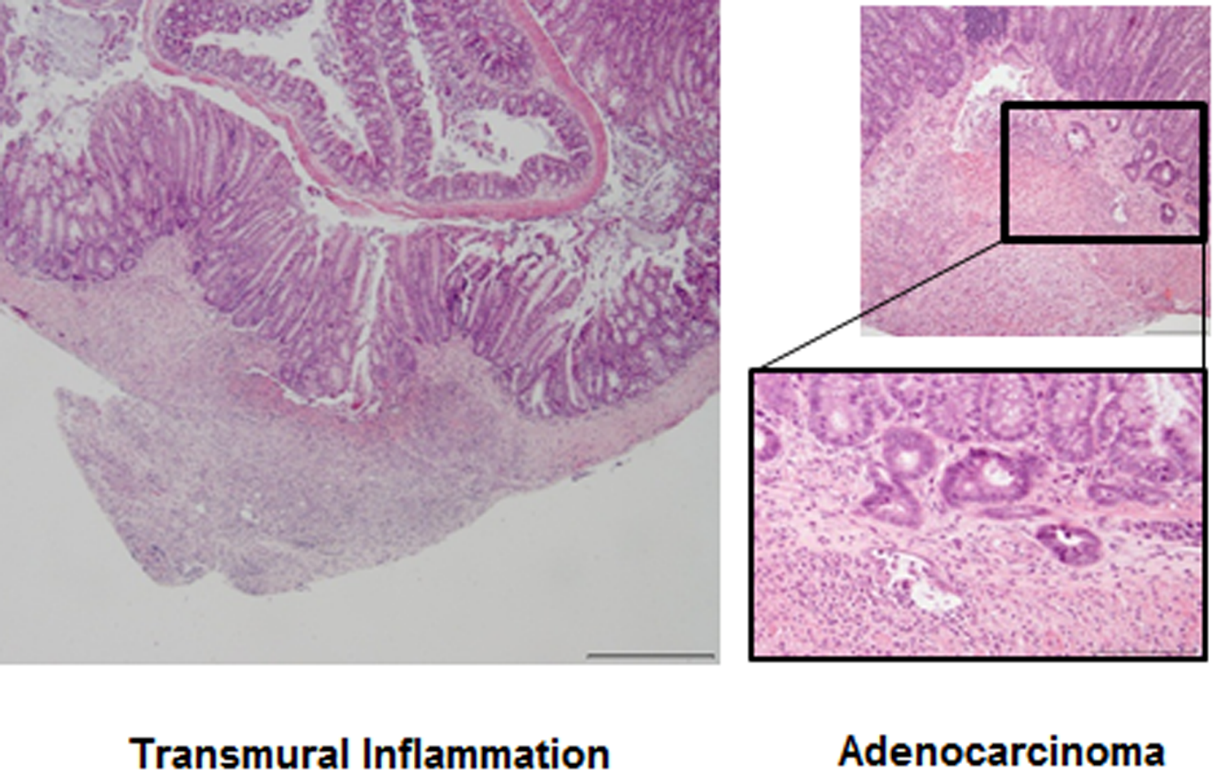


**Figure S6. Transmural inflammation and early invasive adenocarcinoma observed in some surviving Gab2/3^-/-^ mice.** Bone marrow isolated from WT or Gab2/3^-/-^ mice was injected into lethally irradiated WT mice (1 x 10^7^ BM cells/recipient). Representative phenotypes were observed in surviving mice lacking Gab2/3. WT control mice did not show any signs of pathology (data not shown).


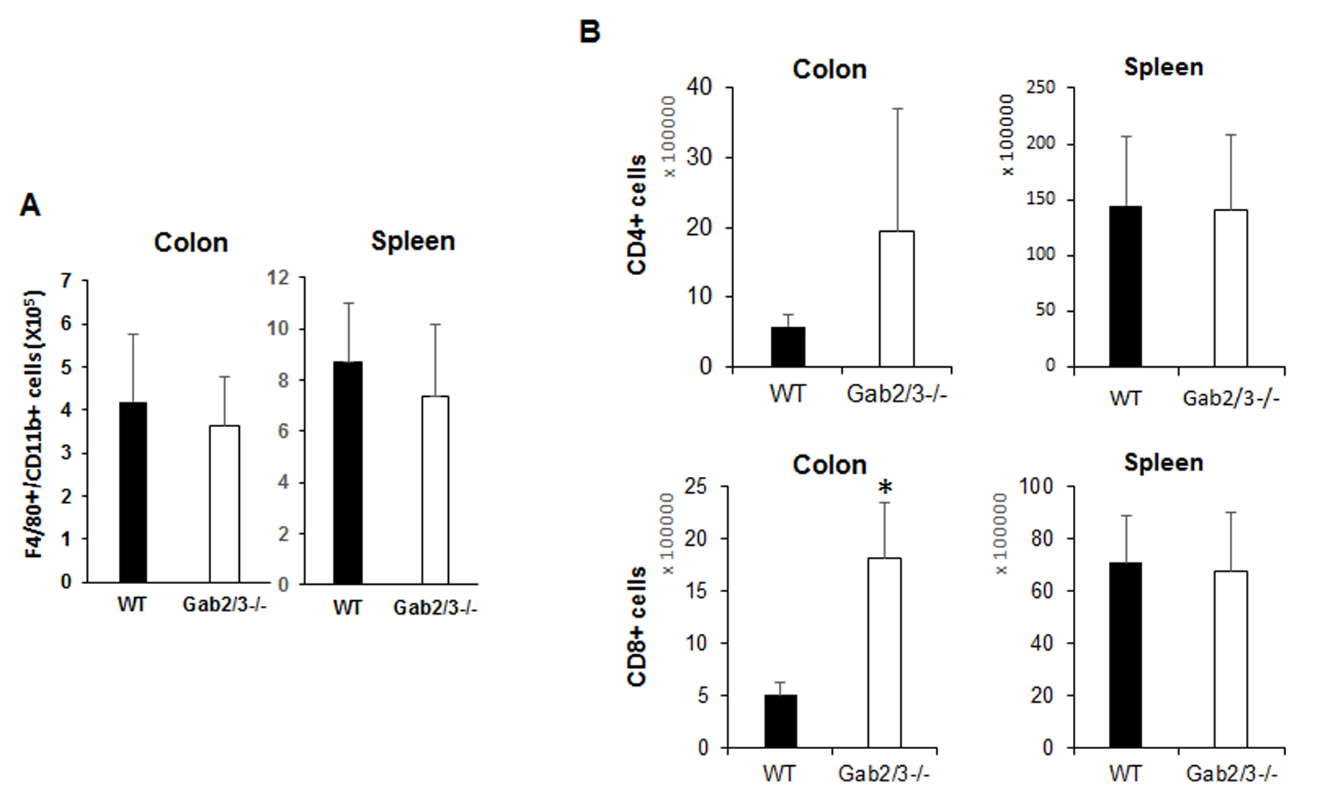


**Figure S7. Absolute number of CD4^+^, CD8^+^, and F4/80^+^CD11b^+^ cells in WT and Gab2/3^-/-^ colon and spleen.** Dead cells were excluded using the LIVE/DEAD fixable aqua dead cells stain kit (Invitrogen, Carlsbad, CA). Antigen presenting cells (APCs) were selected by MHCII and CD45 double staining, and further F4/80^+^CD11b^+^ cells were gated on APCs. Data were acquired with a LSRII flow cytometer (BD Biosciences, San Jose, CA) and analyzed with FlowJo Software (FlowJo, LLC. Ashland, OR). **a)** F4/80^+^CD11b^+^ cells are graphed as absolute numbers in colon and spleen (N=4). **b**) Total CD4^+^ and CD8^+^ cells after colon digestion with collagenase and in mouse splenocytes (N=4, unpaired t-test, * indicated P<0.05).


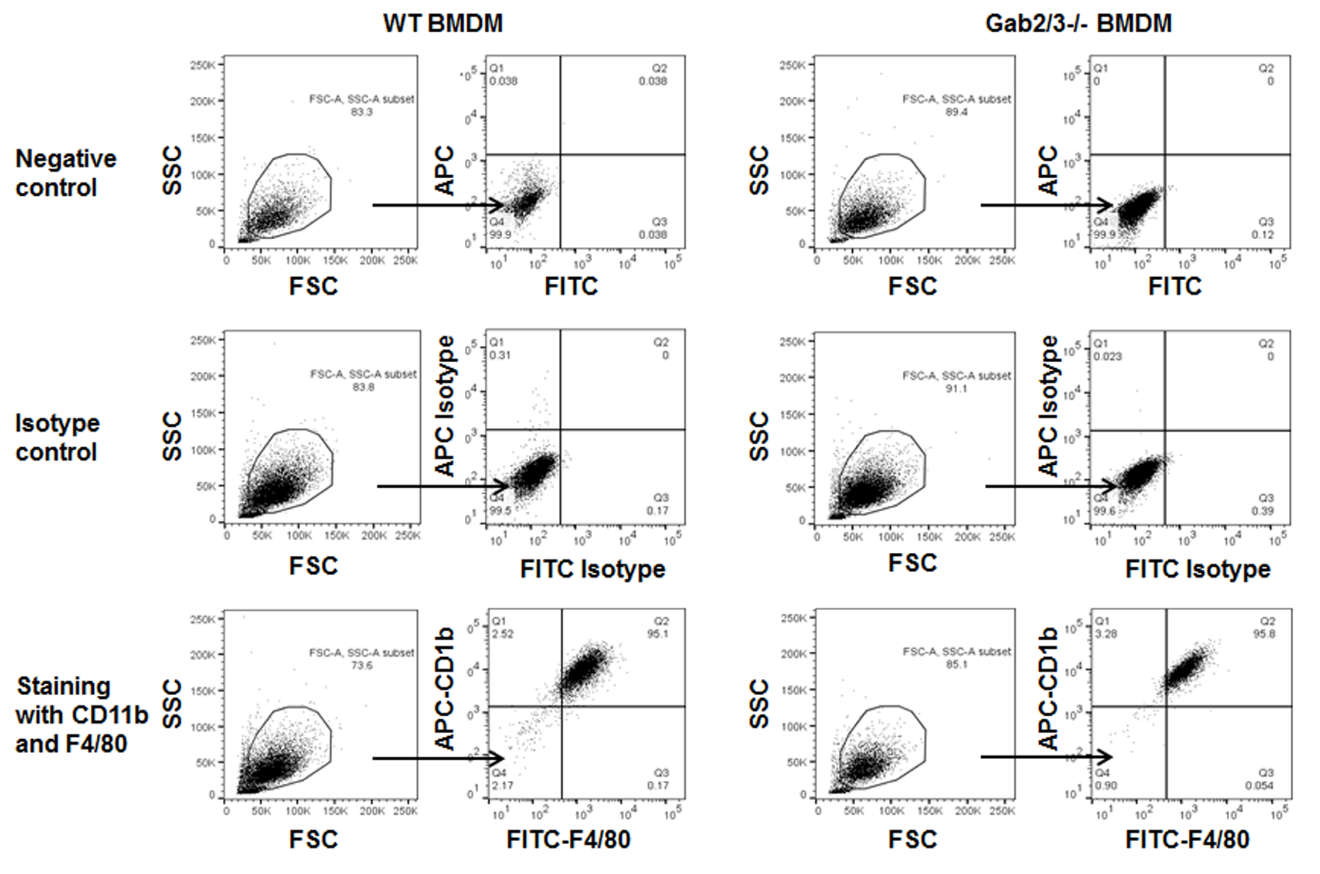


**Figure S8. Representative FACS plots to check the purity of wild-type (WT) and Gab2/3^-/-^ bone marrow-derived macrophages (BMDMs).** WT or Gab2/3^-/-^ BMDMs were first pre-incubated with purified anti-CD16/CD32 antibody (2.4G2) to block non-specific binding for 5-10 minutes on ice (negative control), then stained with either FITC-F4/80 antibody plus APC-CD11b antibody (staining with CD11b and F4/80) or with isotype control (Rat IgG2a, κ Isotype Control, FITC and Rat IgG2b, κ Isotype Control, APC) for 30 minutes at 4 °C. Representative FACS plots are shown. The purity of BMDMs was >95%.


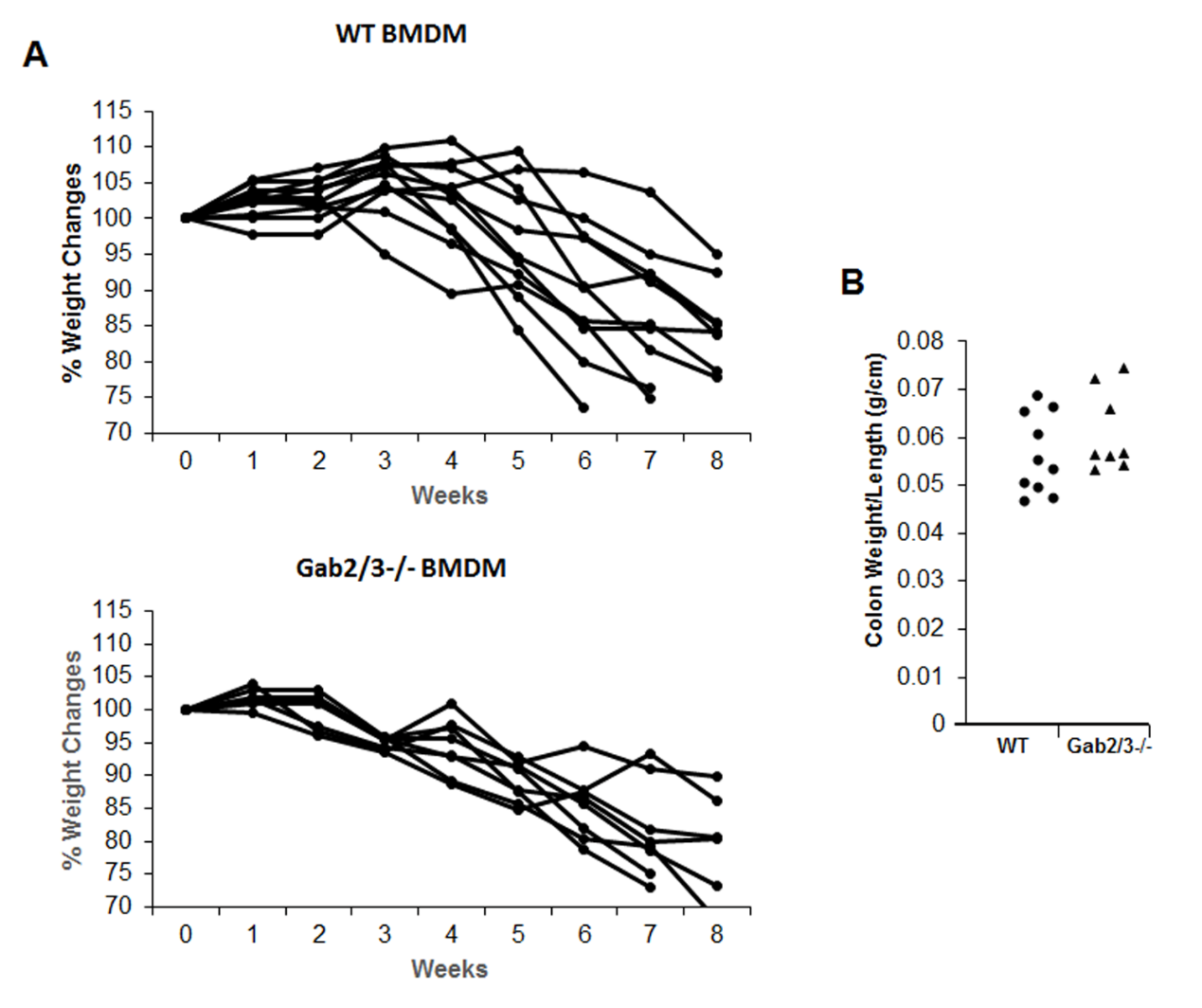


**Figure S9: Mouse weight changes and colon weight/length ratio after transfer of WT naïve CD4^+^ T-cells with either WT or Gab2/3^-/-^ BMDM cells.** A total of 1 x10^6^ LPS-activated WT or Gab2/3^-/-^ BMDMs were transferred into Rag2^-/-^ mice by IP injection and 24 hours later each Rag2^-/-^ mouse was injected with 8 x 10^5^ sorted WT naïve CD4^+^ T-cells. The weight change was monitored weekly. Each line is tracking one individual mouse weight. The results shown are representative of two independent experiments. **b**) The ratio of colon weight/length is shown and was measured upon euthanasia (N=8-10).


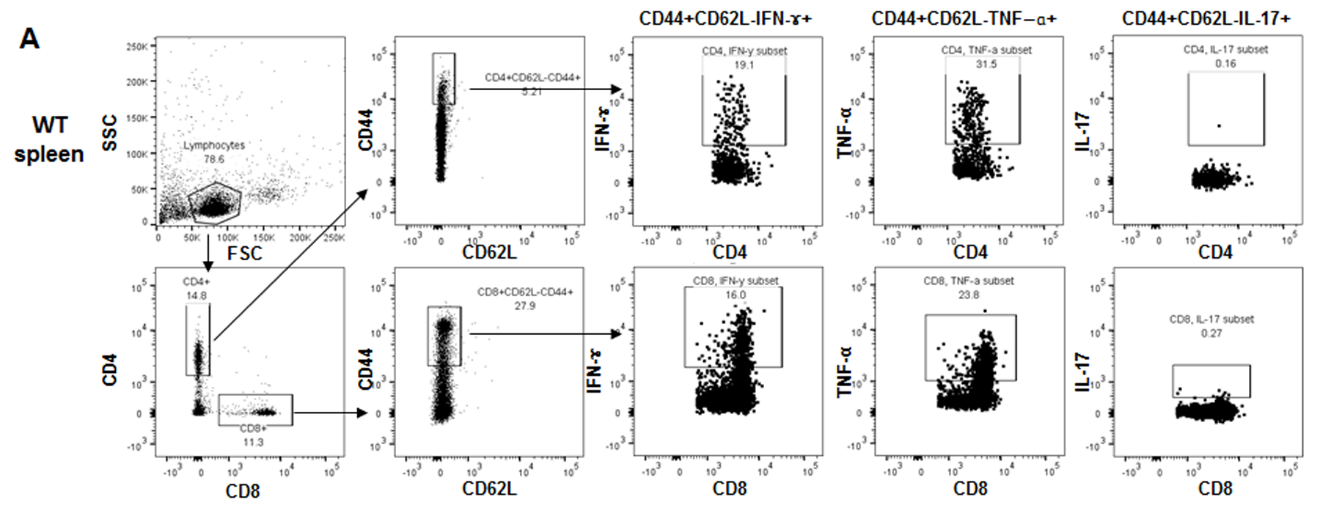

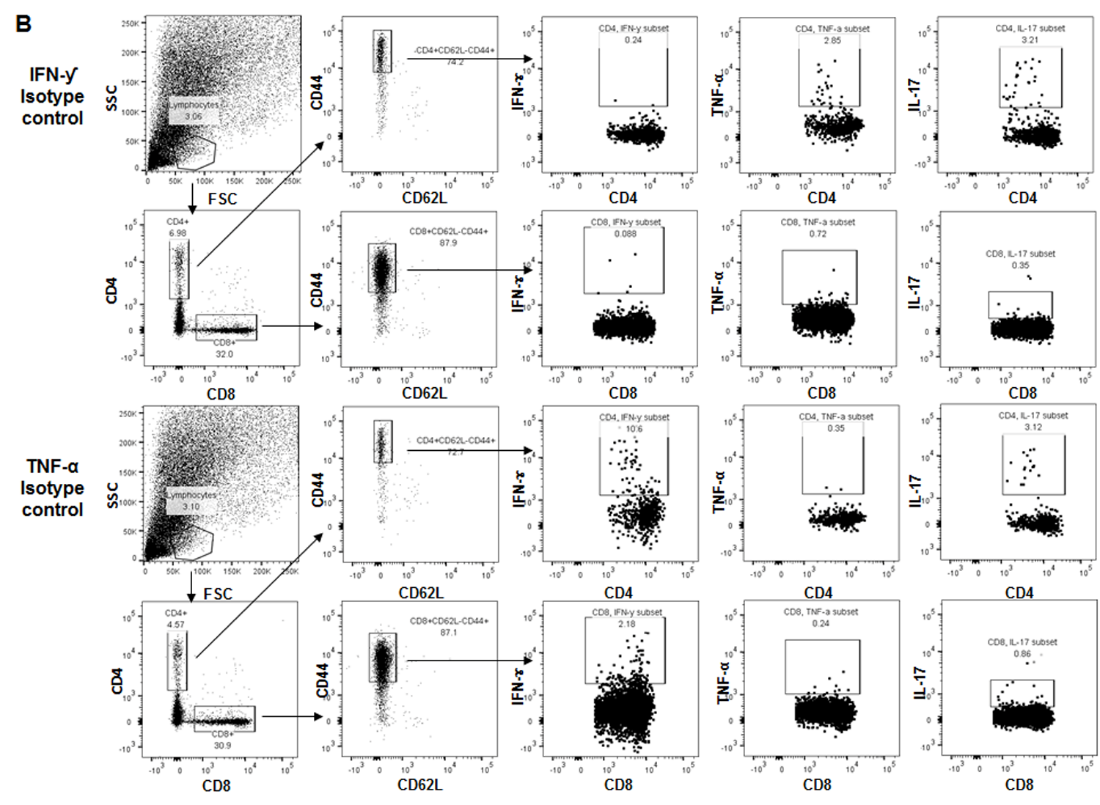

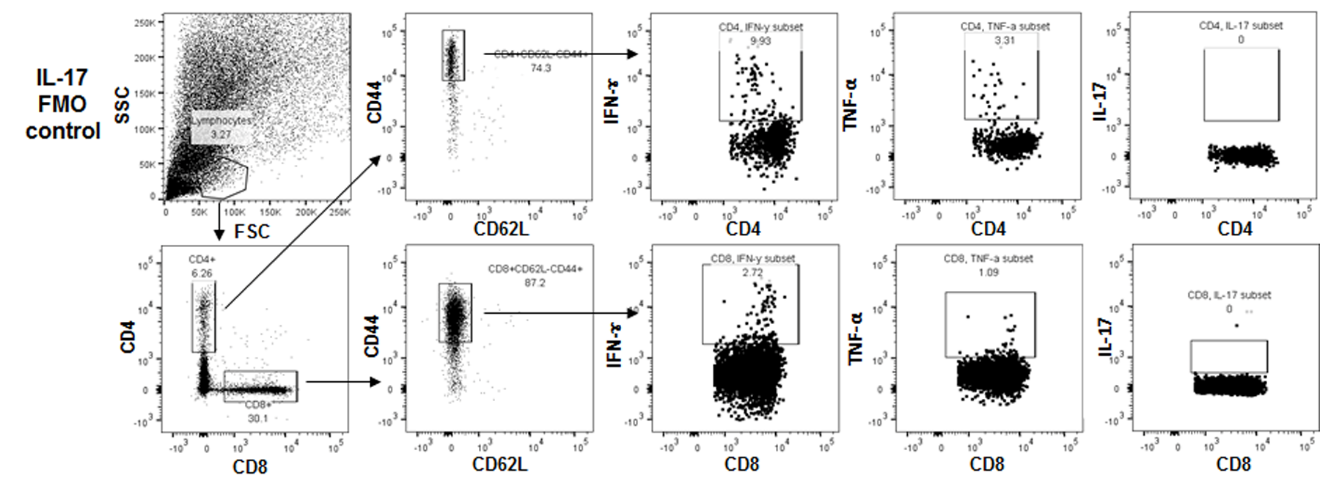


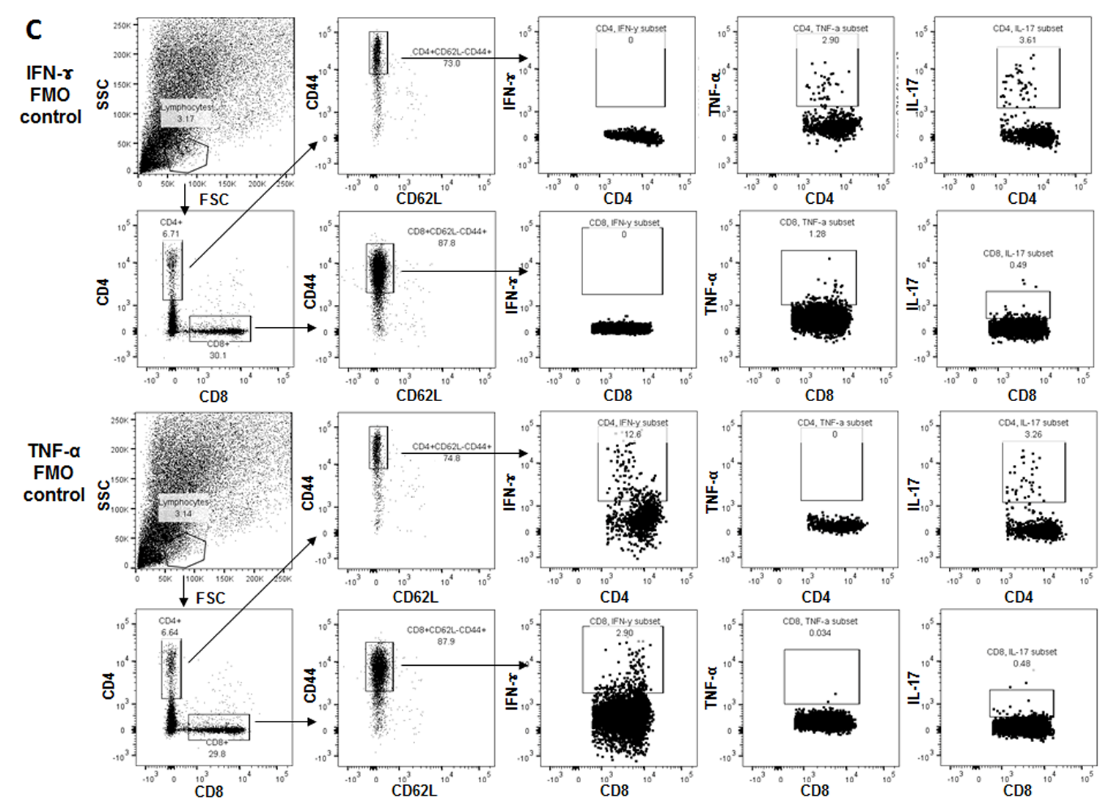
**
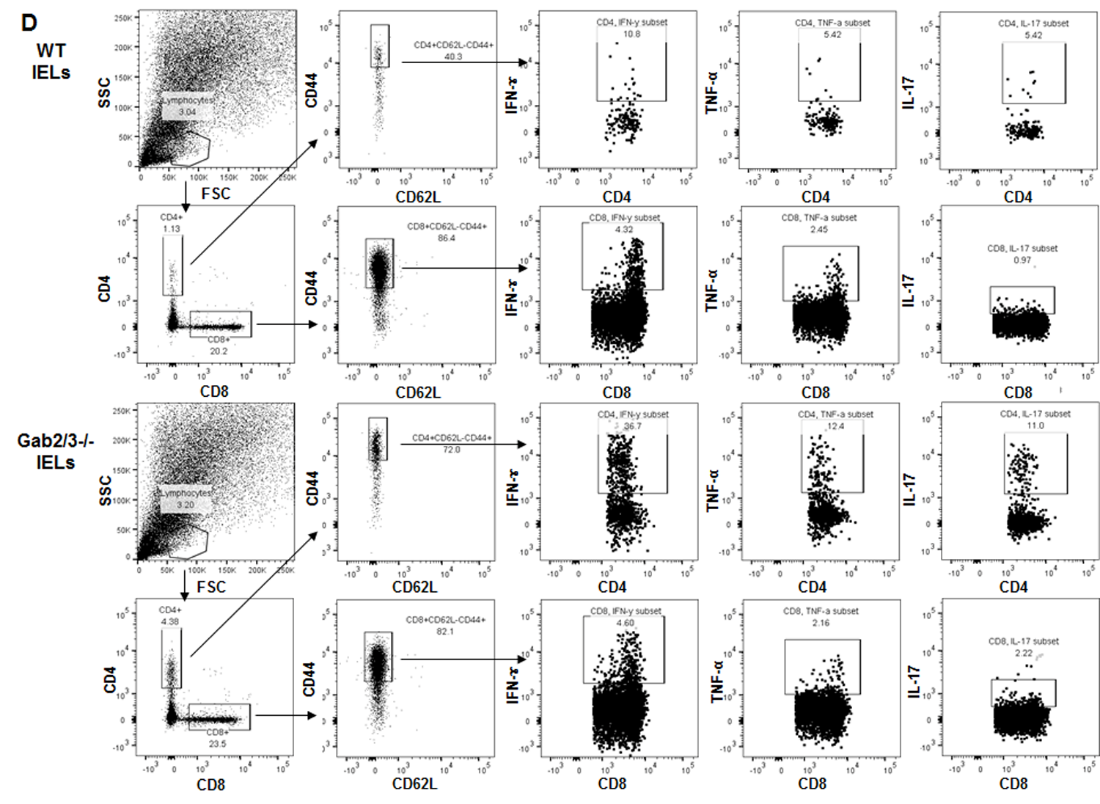
**

**Figure S10. Representative FACS plots to detect IFN-γ, TNF-α, and IL-17 expression in intraepithelial lymphocytes from WT and Gab2/3^-/-^ mice.** Freshly isolated IELs and splenocytes were cultured with PMA, ionomycin, and brefeldin A for 4 hours as described in Materials and Methods. Cells were collected and stained for detection of IFN- γ, TNF-α, and IL-17 secreting CD4^+^CD44^+^CD62L^-^ and CD8^+^CD44^+^CD62L^-^ cells. Cells were gated on CD4^+^, CD8^+^, CD4^+^CD44^+^CD62L^-^, and CD8^+^CD44^+^CD62L^-^ populations before gating on different cytokine-secreting populations. **a**) Cultured wild-type splenocytes were included as controls for gating the lymphocytes population. **b**) The IFN-γ and TNF-α isotype controls were used during cell staining and gating. **c**) Fluorescence minus one (FMO) controls were used during cell staining and gating. **d**) Representative FACS plots for analyzing cytokine secreting WT and Gab2/3^-/-^ IELs. Large dots were used to show the cytokine-secreting populations more clearly.

**
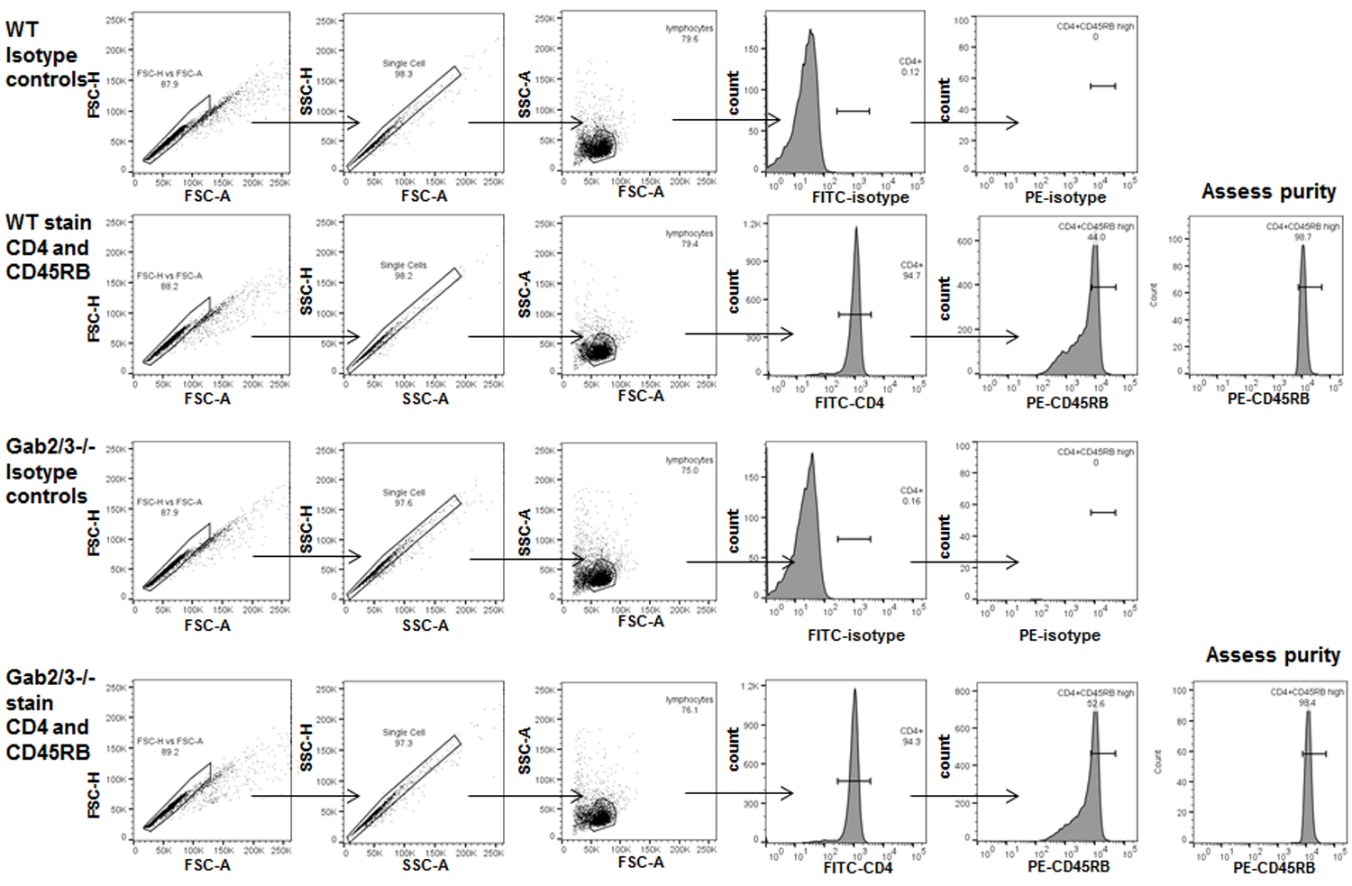

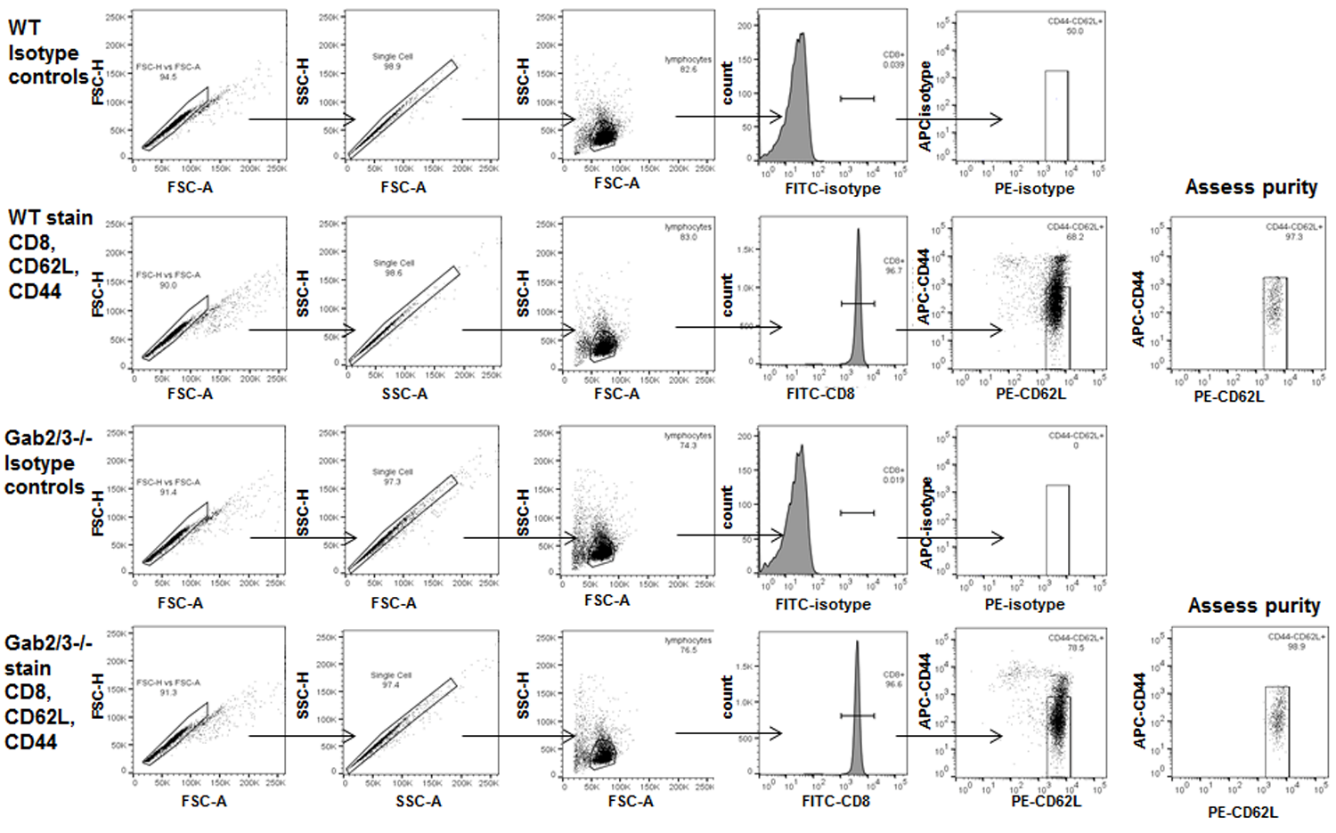
**

**Figure S11. Representative FACS plots for sorting of naïve CD4^+^ and CD8^+^ T-cells a).** Top. Naïve CD4^+^ cells from WT or Gab2/3^-/-^ enriched CD4^+^ T-cells were sorted using the gates shown. Enriched CD4^+^ cells were stained with either CD4^+^ CD45RB or matched isotype controls. An aliquot of each sorted population was run to assess purity (>98%). **b)**. Bottom. Naïve CD8^+^ T-cells from WT or Gab2/3^-/-^ enriched CD8^+^ cells were sorted using the gates shown. Enriched CD8^+^ T-cells were stained with CD8, CD62L, and CD44 or matched isotype controls. Antibody details are listed in Table S1.


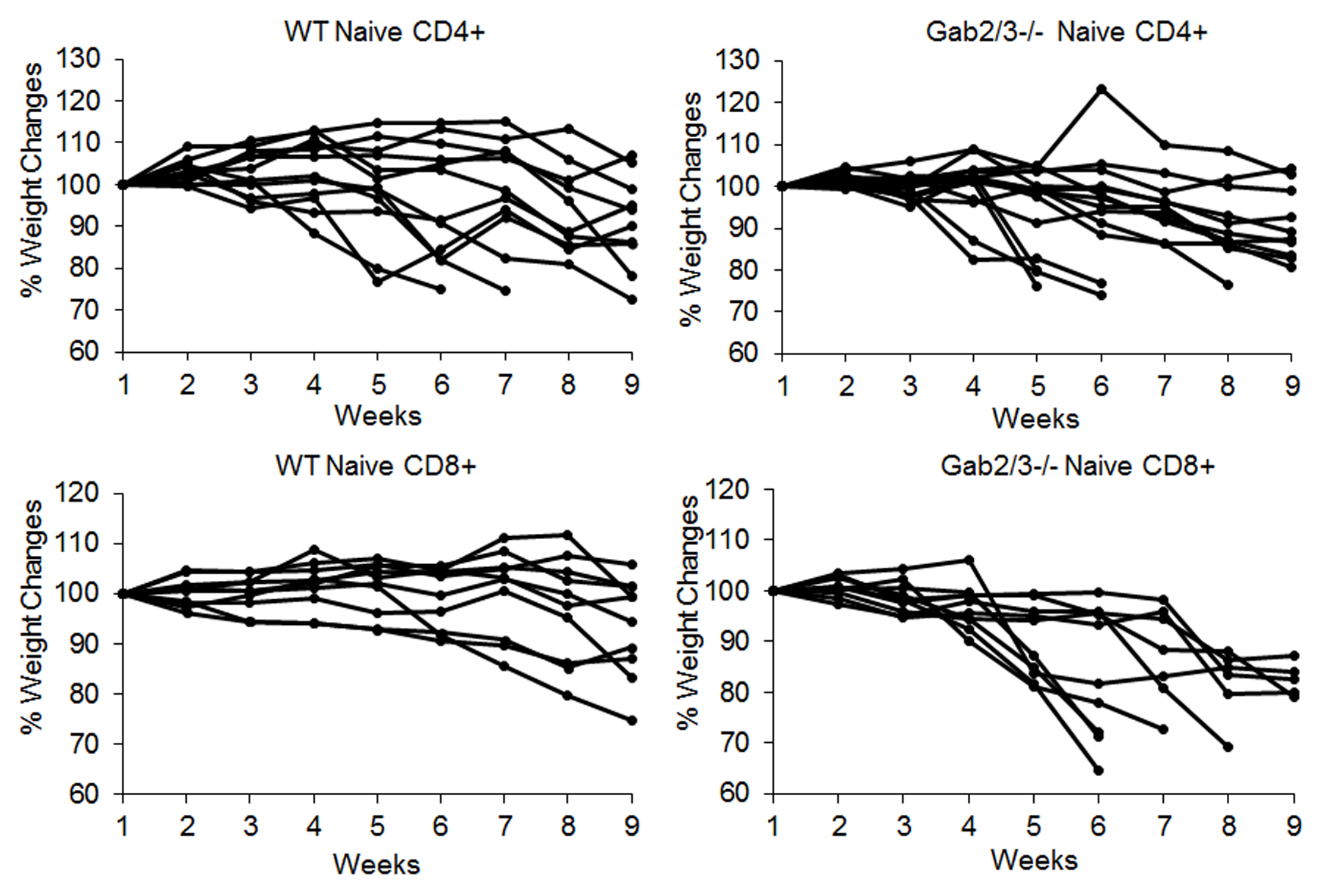


**Figure S12. Mouse weight changes after transfer of WT or Gab2/3^-/-^ naïve CD4^+^ or CD8^+^ T-cells.** FACS sorted naïve CD4^+^ (CD4^+^CD45RB^high^) cells (8x10^5^ cells/recipient) or naïve CD8^+^ (CD8^+^CD44^-^CD62L^+^) cells (4 x10^5^ cells/recipient) from wild-type or Gab2/3^-/-^ mice were transferred into Rag2^-/-^ mice via IP injection. The weight change was monitored weekly. Each line tracks the weight of one individual mouse. Results are combined from two independent experiments.

**
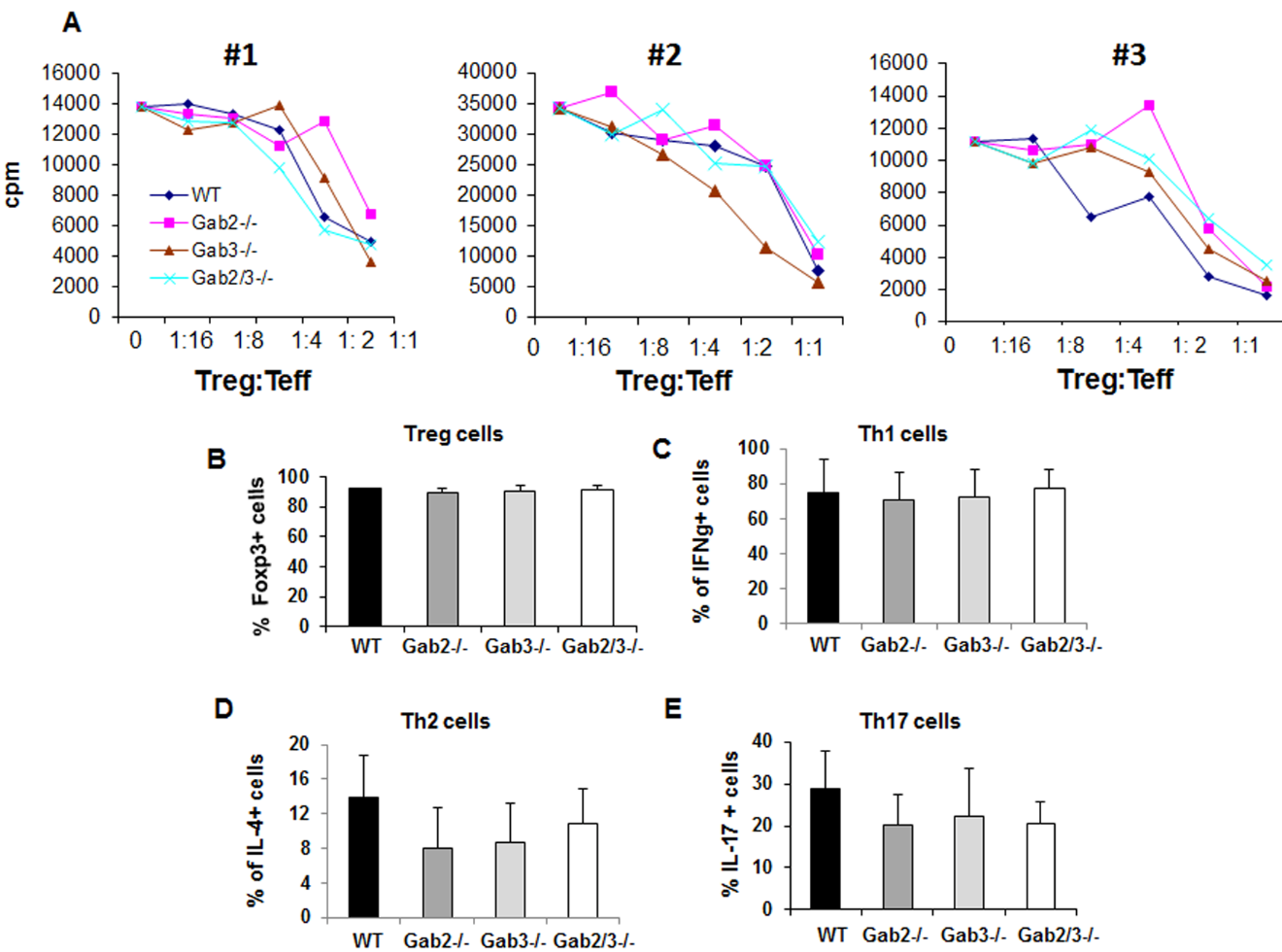
**

**Figure S13. *In vitro* Treg suppressor activity and CD4^+^ T-cell *in vitro* differentiation.** **a)**. *In vitro* Treg suppressor activity results are from three independent sets of mice. Four major CD4^+^ T-cell subsets; Treg, Th1, Th2, and Th17 cells were differentiated from naïve CD4^+^ T-cells upon ligation of their T-cell receptors with antigens depending on the cytokines they received as described in Supplemental Materials and Methods. **b)** Treg differentiation. **c)** Th1 cell differentiation. **d)** Th2 cell differentiation. **e)** Th17 cell differentiation.

**
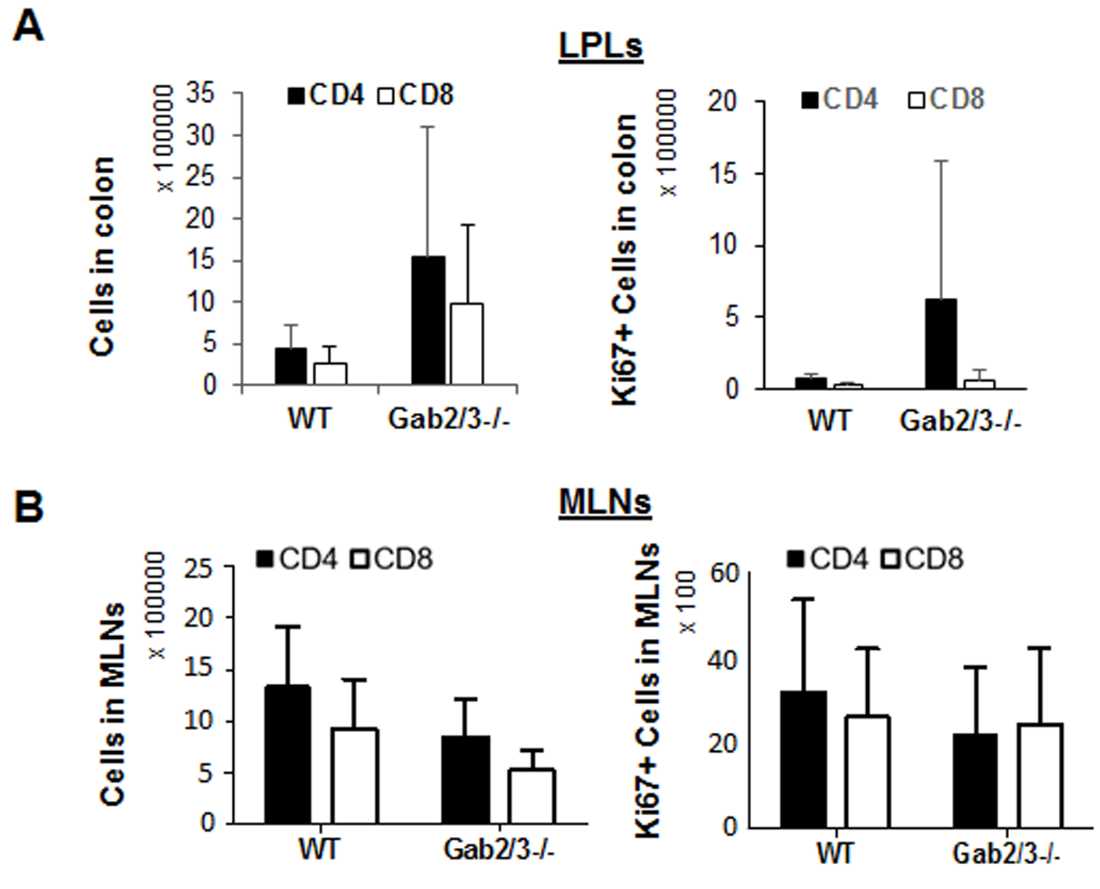
**

**Figure S14. The total number of CD4^+^ and CD8^+^ cells expressing Ki67 in the peripheral lymphoid organs**. Lamina propria of mouse colon and mesenteric lymph nodes were examined (N=5). **a**) The total number of CD4 or CD8 cells and the total number of CD4^+^ or CD8^+^ T-cells expressing Ki67 in the lamina propria of WT or Gab2/3^-/-^ mouse colon. **b**) The total number of CD4^+^ or CD8^+^ cells and the total number of CD4^+^ or CD8^+^ T-cells expressing Ki67 in the lamina propria of WT or Gab2/3^-/-^ mesenteric lymph nodes (MLNs).


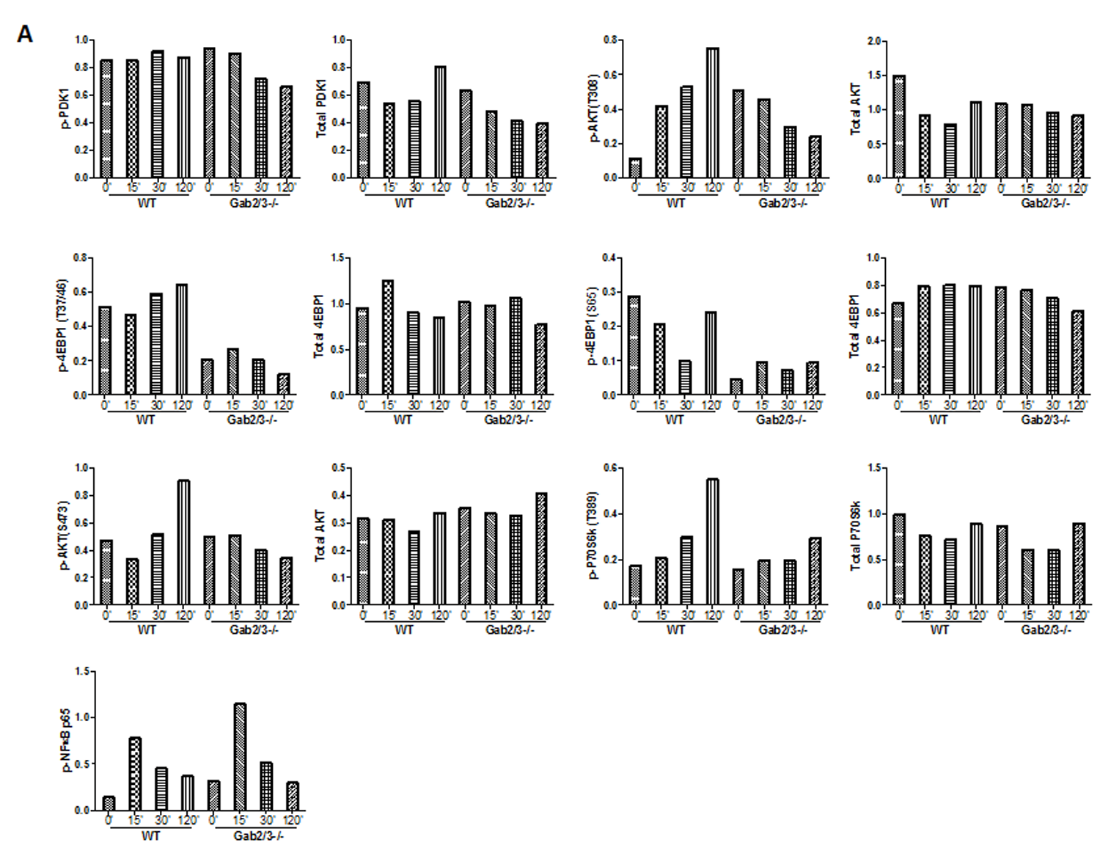

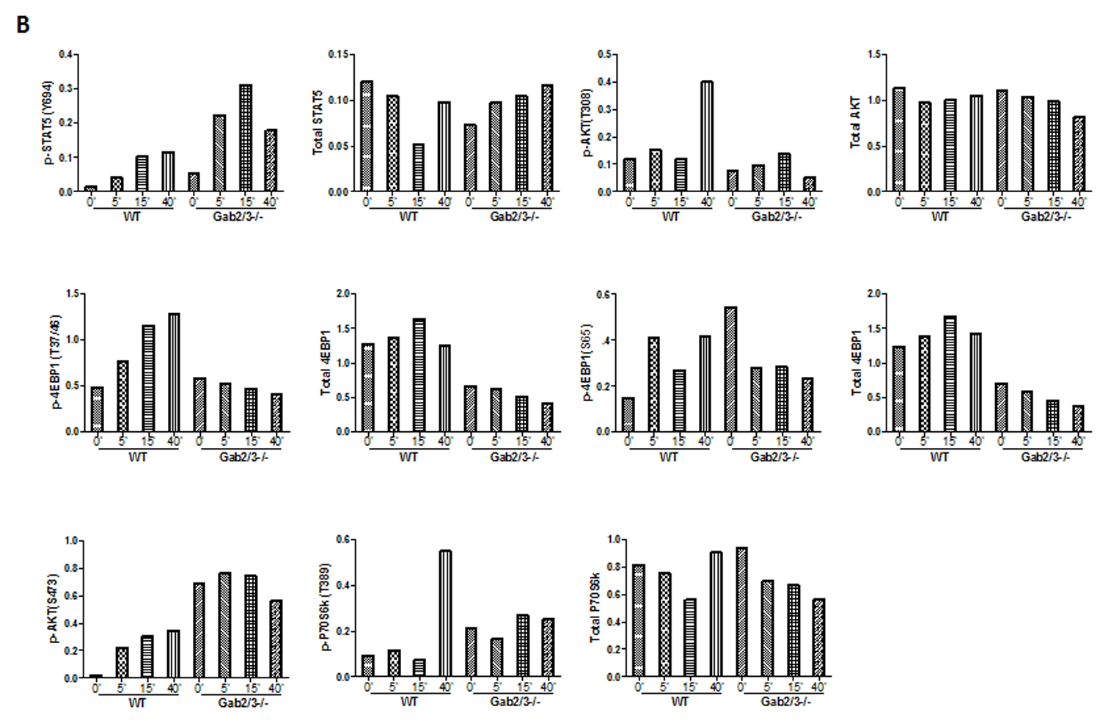


**Figure S15. Western blot densitometry data.** Western blot results from samples: **a**) LPS stimulated BMDM cells and **b**) IL-2 stimulated T-cells were quantified by ImageJ software. All comparisons shown between phospho and total proteins were made relative to the Actin control within the same blot.

**Table S1. Catalog and Clone Numbers for FACS and Western blot Antibodies**

| Name | Clone number | Catalog number | Company |
| --- | --- | --- | --- |
| **Antibodies used for sorting naïve CD4+ and naïve CD8+ cells** | | | |
| CD4 Monoclonal antibody, FITC | GK1.5 | 11-0041-85 | eBiosciences |
| CD45RB antibody, PE | C363.16A | 12-0455-83 | eBiosciences |
| CD8a Monoclonal antibody, FITC | 53-6.7 | 11-0081-82 | Thermo Fisher |
| CD62L (L-Selectin) Monoclonal Antibody, PE | MEL-14 | 12-0621-82 | Thermo Fisher |
| CD44 Monoclonal antibody, APC | IM7 | 17-0441-82 | Thermo Fisher |
| Rat IgG2b, κ Isotype Control, FITC | A95-1 | 556923 | BD Biosciences |
| Rat IgG2a, κ Isotype Control, PE | R35-95 | 553930 | BD Biosciences |
| Rat IgG2b, κ Isotype Control, APC | A95-1 | 553991 | BD Biosciences |
| Purified Rat Anti-Mouse CD16/CD32 | 2.4G2 | 553141 | BD Biosciences |
|  |  |  |  |
| **Antibodies used to check the purity of BMDMs** | | | |
| F4/80 antibody, FITC | BM8 | 123107 | Biolegend |
| CD11b antibody, APC | M1/70 | 101212 | Biolegend |
| Rat IgG2a, κ Isotype Control, FITC | R35-95 | 553929 | BD Biosciences |
| Rat IgG2b, κ Isotype Control, APC | A95-1 | 553991 | BD Biosciences |
|  |  |  |  |
| **Antibodies used to check multilineage donor hematopoietic engraftment** | | | |
| CD45.1 Monoclonal antibody, PE-Cy7 | A20 | 25-0453-82 | eBiosciences |
| CD45.2 antibody, APC | 104 | 109814 | Biolegend |
| Ly-6G/Ly-6C Monoclonal antibody, FITC | RB6-8C5 | 11-5931-85 | eBiosciences |
| TER-119 Monoclonal antibody, FITC | Ter-119 | 11-5921-82 | Thermo Fisher |
| CD45R (B220) Monoclonal antibody, eFluor 450 | RA3- 6B2 | 48-0452-82 | eBiosciences |
| CD4 Monoclonal antibody, eFluor 450 | GK1.5 | 48-0041-82 | Thermo Fisher |
| Rat Anti-Mouse CD8a, PE | 53-6.7 | 553032 | BD Biosciences |
|  |  |  |  |
| **Antibodies used to analyze macrophage and T-cells in splenocytes or digested colon** | | | |
| LIVE/DEAD™ Fixable Aqua Dead Cell Stain Kit |  | L34957 | Invitrogen |
| PerCP Rat Anti-Mouse CD45 | 30-F11 | 557235 | BD Biosciences |
| MHC Class II (I-A/I-E), Alexa Fluor 700 | M5/114.15.2 | 56-5321-82 | eBiosciences |
| CD11b Monoclonal antibody, eFluor 450 | M1/70 | 48-0112-82 | eBiosciences |
| F4/80 Monoclonal antibody, PE-Cy7 | BM8 | 25-4801-82 | eBiosciences |
| Rat Anti-Mouse CD8a, PE | 53-6.7 | 553032 | BD Biosciences |
| CD4, APC | RM4-5 | 17-0042-83 | eBiosciences |
|  |  |  |  |
| **Antibodies used to analyze subpopulations of splenic T-cells** | | | |
| CD4 Monoclonal antibody, eFluor 450 | GK1.5 | 48-0041-82 | Thermo Fisher |
| BV650 anti-mouse CD8a antibody | 53-6.7 | 100742 | Biolegend |
| CD62L (L-Selectin) Monoclonal antibody, PE | MEL-14 | 12-0621-82 | Thermo Fisher |
| CD44 Monoclonal antibody, APC | IM7 | 17-0441-82 | Thermo Fisher |
| PerCP/Cy5.5 anti-mouse CD49d antibody | R1-2 | 103619 | Biolegend |
| FITC anti-mouse CD122 (IL-2Rβ) antibody | TM-β1 | 123208 | Biolegend |
|  |  |  |  |
| **Antibodies used for intracellular staining of IFN-γ, TNF-α, and IL-17A** | | | |
| PE anti-mouse IFN-y antibody | XMG1.2 | 505807 | Biolegend |
| FITC anti-mouse TNF-α antibody | MP6-XT22 | 506303 | Biolegend |
| PerCP/Cy5.5 anti-mouse IL-17A antibody | TC11-18H10.1 | 06919 | Biolegend |
| Mouse anti-Ki67, BV421 | B56 | 562899 | BD Biosciences |
| BV650 anti-mouse CD8a antibody | 53-6.7 | 100742 | Biolegend |
| PE-Cy™7 Rat Anti-Mouse CD4 | GK1.5 | 563933 | BD Biosciences |
| CD62L (L-Selectin) Monoclonal antibody, PE | MEL-14 | 12-0621-82 | Thermo Fisher |
| CD44 Monoclonal antibody, APC | IM7 | 17-0441-82 | Thermo Fisher |
| Purified NA/LE Hamster Anti-Mouse CD28 | 37.51 | 553294 | BD Biosciences |
| CD3e Monoclonal antibody | 145-2C11 | 16-0031-82 | Thermo Fisher |
| Anti-mouse/human CD11b antibody, APC | M1/70 | 101212 | Biolegend |
| F4/80 Monoclonal antibody, PE-Cy7 | BM8 | 553032 | eBiosciences |
|  |  |  |  |
| **Antibodies used for Western blot analysis** | | | |
| T-PDK-1 |  | 3062 | Cell signaling |
| Phospho-PDK1 (S241) | C49H2 | 3438 | Cell signaling |
| Phospho-AKT (T308) | D25E6 | 13038 | Cell signaling |
| Phospho-AKT (S473) | D9E | 4060 | Cell signaling |
| Phospho-4EBP1 (T37/46) | 236B4 | 2855S | Cell signaling |
| Phospho-4EBP1 (65S) |  | 9451S | Cell signaling |
| T-4EBP1 | 53H11 | 9644 | Cell signaling |
| Phospho-P70S6K (T389) | 108D2 | 9234 | Cell signaling |
| T-P70S6K |  | 9202S | Cell signaling |
| Phospho-STAT5 |  | 9351S | Cell signaling |
| AKT antibody |  | 9272 | Cell signaling |
| Purified mouse anti-STAT5 monoclonal antibody | 89/Stat5 | 610191 | BD Biosciences |
| Monoclonal Anti-ß-Actin antibody | AC-15 | A5441 | Sigma-Aldrich |
| Anti-Glyceraldehyde-3-Phosphate Dehydrogenase antibody | 6C5 | MAB374 | Millipore Sigma |
| p-NF-ĸB p65 (S536) | 93H1 | 3033S | Cell signaling |
| IRDye 680RD Goat anti-mouse IgG (H+L) |  | 925-68070 | LI-COR |
| IRDye 800CW Goat anti-Rabbit IgG (H+L) |  | 925-32211 | LI-COR |

**Table S2. Comprehensive Diagnostic Profile of WT and Gab2/3^-/-^ Mouse Serum**

|  | Albumin | Alk phos | ALT | Amylase | TBILI | BUN | Calcium |
| --- | --- | --- | --- | --- | --- | --- | --- |
|  | g/dl | U/L | U/L | U/L | mg/dL | mg/dL | mg/dL |
| WT (N=6) | 4.3 ± 0.02 | 103.2 ± 34.4 | 34.3 ± 6.9 | 814 ± 81 | 0.3 ± 0.1 | 34.0 ± 2.2 | 10.3 ± 0.3 |
| Gab2/3-/- (N=6) | 3.4 ± 0.7* | 94.2 ± 71.9 | 40.2 ± 11.4 | 1127 ± 219** | 0.3 ± 0.1 | 9.7 ± 0.6 | 6.6 ± 1.4 |

|  | Phosphorus | Creatine | Glucose | Na+ | K+ | Total Protein | Globulin |
| --- | --- | --- | --- | --- | --- | --- | --- |
|  | mg/dL | mg/dL | mg/dL | mmol/L | mmol/L | g/dl | g/dl |
| WT (N=6) | 7.4 ± 1.1 | 0.2 ± 0.1 | 142.5 ± 20.1 | 151 ± 1.9 | 6.2 ± 0.8 | 5.6 ± 0.1 | 1.3 ± 0.4 |
| Gab2/3-/- (N=6) | 6.6 ± 1.4 | 0.3 ± 0.1 | 133.3 ± 25.1 | 148 ± 3.6 | 5.3 ± 1.2 | 5.1 ± 0.5 | 1.7 ± 0.2* |

**Notes:** *P ≤ 0.05; ** P ≤ 0.01 (unpaired t-test); Alk: Alkaline; ALT: Alanine Aminotransferase; TBILI: Total Bilirubin;

BUN: Blood Urea Nitrogen

Supplemental References

Sekiya, T., and A. Yoshimura. 2016. In Vitro Th Differentiation Protocol. *Methods Mol Biol* 1344:183-191.

Zeng, H., Y. Chen, M. Yu, L. Xue, X. Gao, S.W. Morris, D. Wang, and R. Wen. 2008. T cell receptor-mediated activation of CD4+CD44hi T cells bypasses Bcl10: an implication of differential NF-kappaB dependence of naive and memory T cells during T cell receptor-mediated responses. *J Biol Chem* 283:24392-24399.
